# Type VI secretion systems of plant‐pathogenic *Burkholderia glumae* BGR1 play a functionally distinct role in interspecies interactions and virulence

**DOI:** 10.1111/mpp.12966

**Published:** 2020-07-09

**Authors:** Namgyu Kim, Jin Ju Kim, Inyoung Kim, Mohamed Mannaa, Jungwook Park, Juyun Kim, Hyun‐Hee Lee, Sais‐Beul Lee, Dong‐Soo Park, Woo Jun Sul, Young‐Su Seo

**Affiliations:** ^1^ Department of Integrated Biological Science Pusan National University Busan Korea; ^2^ Department of Systems Biotechnology Chung‐Ang University Anseong Korea; ^3^ National Institute of Crop Science Milyang Korea

**Keywords:** bacterial competition, bacterial virulence, *Burkholderia glumae*, interspecies interaction, metagenomic analysis, TssD, type VI secretion system

## Abstract

In the environment, bacteria show close association, such as interspecies interaction, with other bacteria as well as host organisms. The type VI secretion system (T6SS) in gram‐negative bacteria is involved in bacterial competition or virulence. The plant pathogen *Burkholderia glumae* BGR1, causing bacterial panicle blight in rice, has four T6SS gene clusters. The presence of at least one T6SS gene cluster in an organism indicates its distinct role, like in the bacterial and eukaryotic cell targeting system. In this study, deletion mutants targeting four *tssD* genes, which encode the main component of T6SS needle formation, were constructed to functionally dissect the four T6SSs in *B. glumae* BGR1. We found that both T6SS group_4 and group_5, belonging to the eukaryotic targeting system, act independently as bacterial virulence factors toward host plants. In contrast, T6SS group_1 is involved in bacterial competition by exerting antibacterial effects. The *Δ* *tssD1* mutant lost the antibacterial effect of T6SS group_1. The Δ*tssD1* mutant showed similar virulence as the wild‐type BGR1 in rice because the Δ*tssD1* mutant, like the wild‐type BGR1, still has key virulence factors such as toxin production towards rice. However, metagenomic analysis showed different bacterial communities in rice infected with the Δ*tssD1* mutant compared to wild‐type BGR1. In particular, the T6SS group_1 controls endophytic plant‐associated bacteria such as *Luteibacter* and *Dyella* in rice plants and may have an advantage in competing with endophytic plant‐associated bacteria for settlement inside rice plants in the environment. Thus, *B. glumae* BGR1 causes disease using T6SSs with functionally distinct roles.

## Graphical abstract

T6SS group_4 and T6SS group_5 affect the pathogenicity of *Burkholderia glumae* BGR1 as a completely independent eukaryotic targeting system, whereas T6SS group_1 is involved in bacterial competition by an antibacterial effect.

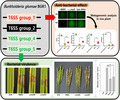

## 1 INTRODUCTION

Myriad bacteria demonstrate close associations with nearby bacteria, as well as with their host organisms (Freestone, 2013; Stubbendieck *et al*., 2016). Bacteria can persist and adapt in their ecological niche by continuously interacting with other organisms, and have developed many protective and infectious mechanisms against competitors and host organisms. Some of these, such as bacterial secretion systems, biofilms, and phytotoxins, provide survival benefits against bacterial and eukaryotic cells, and facilitate environmental adaptation (Backert and Meyer, 2006). In particular, bacterial secretion systems are considered the primary and effective means for communication and adaption to the surroundings (Russell *et al*., 2014).

Bacterial secretion systems are classified into nine groups (Tat system, types I–VII, type IX) according to their structures, functions, and specific effectors (Stanley *et al*., 2000; Whitney *et al*., 2013; Kneuper *et al*., 2014; Green and Mecsas, 2016). The type VI secretion system (T6SS) was identified as a bacterial secretion system in an opportunistic pathogen, *Pseudomonas aeruginosa* (Mougous *et al*., 2006). The T6SS is widespread in around 25% of gram‐negative bacteria and is easily found in pathogenic or symbiotic plant‐associated bacteria (Boyer *et al*., 2009; Bernal *et al*., 2018). The T6SS forms proteinaceous machinery that delivers substrates (effectors) to adjacent bacterial or eukaryotic cells in a contact‐dependent manner or releases effectors tothe extracellular environment. The effectors secreted by these systems are used to destroy and manipulate eukaryotic cells and/or to fight other bacteria to gain dominant status in the same ecological niche (Ma and Mekalanos, 2010). At least one T6SS gene cluster is present in some bacteria. The presence of two T6SS gene clusters in one organism increases the possibility that each system has a different function depending on the circumstances (Bingle *et al*., 2008). More recently, functional T6SSs were discovered in a broad spectrum of bacterial species and were found to contribute to virulence in host organisms, improve bacterial robustness, and enhance adaptation to polymicrobial environments through bacterial competition (Russell *et al*., 2014). For example, *P. aeruginosa* has H1‐T6SS, H2‐T6SS, and H3‐T6SS. H1‐T6SS contributes only to interactions between bacteria by the delivery of toxins and effector molecules (Hood *et al*., 2010). H2‐T6SS and H3‐T6SS also exhibit antibacterial activity by secreting trans‐kingdom effectors, but are involved in virulence against eukaryotes (Russell *et al*., 2013; Jiang *et al*., 2014). T6SS‐5 of *Burkholderia thailandensis* is also a major virulence factor in the murine model of acute melioidosis (Schwarz *et al*., 2010).

The T6SS is composed of a minimal set of 13 core components, TssA–TssM (type six secretion). Apart from these major components, several accessory proteins are encoded in the T6SS gene cluster, and work together with the T6SS effector to damage the inner membrane of prokaryotic target cells or participate in negative regulation of the T6SS (Miyata *et al*., 2013; Lin *et al*., 2018). These components are generally encoded by clustered genes; each cluster has a different gene organization despite the clusters being in a single organism. Among the core components, TssD, also known as Hcp (hemolysin co‐regulated protein), and VgrG (valine‐glycine repeat protein G) have been considered the hallmark apparatus of T6SS as well as secreted effectors in all bacteria with functional T6SSs (Pukatzki *et al*., 2007).

*B. glumae* is a gram‐negative bacterium known as a seedborne phytopathogenic bacterium that causes bacterial panicle blight as well as sheath and seedling rot in rice (*Oryza sativa*) (Goto, 1956; Goto *et al*., 1987). The major virulence factors of *B. glumae* are toxoflavin, lipase, type III effectors, and extracellular polysaccharides, along with cell motility (Iiyama *et al*., 1995; Suzuki *et al*., 2004; Kim *et al*., 2007; Kang *et al*., 2008; Lee *et al*., 2016; Jung *et al*., 2018). The T6SS was classified into six groups based on the distribution of 5 out of 13 T6SS components in 12 *Burkholderia* strains, including *B. glumae* (Seo *et al*., 2015). Of these six T6SS groups, four T6SS groups are found in *B. glumae* BGR1. However, their functions are still unexplored.

In this study, we report that T6SS group_1 has antibacterial effects but is dispensable for virulence, whereas T6SS group_4 and T6SS group_5 were found to directly affect the pathogenicity of *B. glumae* BGR1 targeting rice plants through phylogenetic analysis and a phenotypic assay. Furthermore, metagenomics analysis of the endophytic bacterial community inside plants identified different bacterial communities with the Δ*tssD1* mutant compared to those with the wild‐type BGR1. This indicates that T6SS group_1 inhibits or limits the growth of endophytic plant‐associated bacteria, thereby helping pathogenic bacteria form a bacterial community easily. Our findings clearly describe the important role of T6SS in interspecies interactions, including pathogen–host interaction and interaction with other bacteria.

## 2 RESULTS

### 2.1 Evolutionary analysis of type VI secretion systems in *Burkholderia* species shows functional differentiation based on environmental adaptation

The evolutionary analysis of *Burkholderia* T6SS in *B*. *mallei*, *B. pseudomallei*, *B. thailandensis*, and *B*. *cepacia,* as well as in six rice‐pathogenic strains of *B*. *plantarii*, *B. gladioli*, and *B*. *glumae*, was performed to identify the distinct roles of the four T6SS in *B*. *glumae* BGR1, using at least 10 major core components of T6SS. The T6SS gene clusters of *B. glumae* BGR1, *B. plantarii* ATCC43733, and *B. gladioli* BSR3 were annotated in our previous study (Seo *et al*., 2015). All *Burkholderia* species have at least one T6SS gene cluster. However, we found that not all T6SS gene clusters of *Burkholderia* species were completely composed of 13 major elements. In the resulting phylogeny, six rice‐pathogenic *Burkholderia* species were highly conserved in at least one T6SS gene cluster, which includes T6SS group_1 of *B. glumae*, *B. gladioli*, and *B. plantarii* (Figure S1). These T6SS gene clusters are closely clustered with *B. thailandensis* E264 T6SS‐1, a well‐known bacterial cell targeting system (Schwarz *et al*., 2010). In addition, T6SS group_4 and T6SS group_5 of *B. glumae* BGR1 were clustered with the T6SS gene clusters of the eukaryotic cell targeting system, which are *B. thailandensis* E264 T6SS‐5 and *B. pseudomallei* K96243 T6SS‐5, in one or two subtrees (Figure S1) (Schwarz *et al*., 2010; Lennings *et al*., 2019). T6SS group_2 was clustered in one or two subtrees with T6SS‐2 of *B. pseudomallei* K96243 and *B. thailandensis* E264. However, T6SS group_2 was not clustered with the bacterial and eukaryotic cell targeting systems despite the presence of a complete T6SS gene cluster. Therefore, we assumed that the T6SS of *B. glumae* BGR1 is involved in the bacterial cell targeting system and the eukaryotic cell targeting system through T6SS group_1 and both T6SS group_4 and 5, respectively.

### 2.2 The *tssD* deletion mutants were constructed to evaluate the functions of four T6SS systems in *B. glumae* BGR1

**Figure 1:**
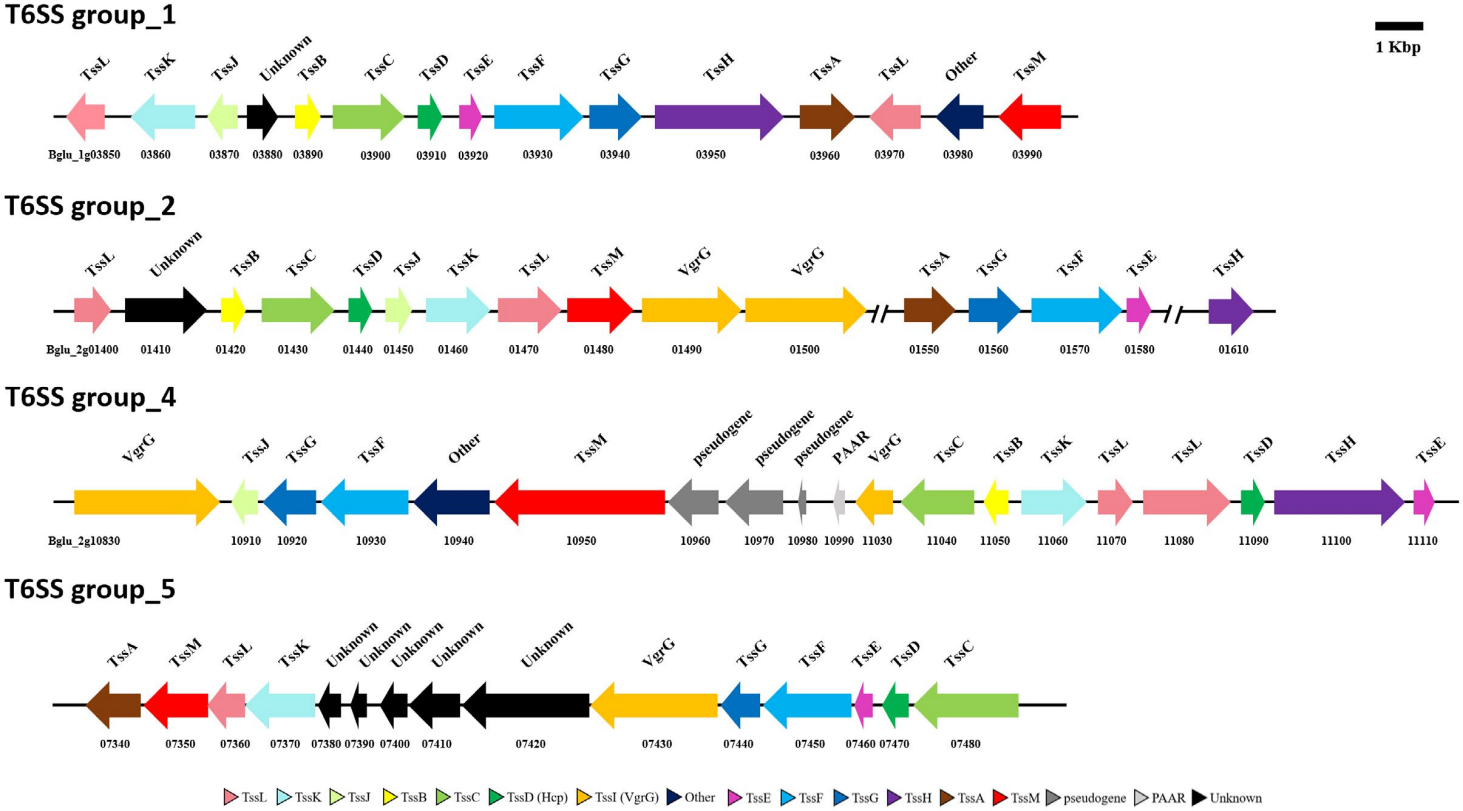
Genetic organization of the four type VI secretion system (T6SS) clusters in *Burkholderia glumae* BGR1. *B. glumae* BGR1 has four T6SS gene clusters in its genome. The genes are indicated by the locus ID (e.g., bglu_1g03850) and are to scale. The COGs of 13 conserved T6SSs from the SecreT6 database (http://db‐mml.sjtu.edu.cn/SecReT6/) are marked with specific colours in the four T6SS clusters of *B. glumae* BGR1 (Li *et al*., 2015)

*B. glumae* BGR1 has four T6SS gene clusters (T6SS group_1, T6SS group_2, T6SS group_4, and T6SS group_5) with different genetic architectures as well as Tss components (Figure 1). The T6SS group_2 and group_4 clusters possess all of the 13 T6SS core components. However, the T6SS group_1 cluster lacks TssI (VgrG) and the T6SS group_5 cluster lacks TssB, TssH, and TssJ. The *tssD1* gene (bglu_1g03910) belongs to T6SS group_1 and is located in chromosome 1. The *tssD2* gene (bglu_2g01440), *tssD4* gene (bglu_2g11090), and *tssD5* gene (bglu_2g07470) are located in chromosome 2 and belong to T6SS group_2, T6SS group_4, and T6SS group_5, respectively. To determine the functions of each T6SS, we constructed markerless deletion mutants targeting the four *tssD* genes via two homologous recombinations. After completion of the second recombination, *tssD* deletion mutant strains were confirmed by PCR (Figure S2 and Table S3). Single deletion mutants called Δ*tssD1*, Δ*tssD2*, Δ*tssD4*, and Δ*tssD5*, corresponding to the genes *tssD1*, *tssD2*, *tssD4*, and *tssD5*, respectively, were generated. Double deletion mutants, Δ*tssD12* and Δ*tssD45*, were generated. In addition, a quadruple deletion mutant called Δ*tssD1245* was also constructed. In order to complement the phenotype of *tssD* deletion mutants Δ*tssD1*, Δ*tssD4*, Δ*tssD5,* and Δ*tssD45*, the respective complementation strains, Δ*tss1D*‐C, Δ*tss4D*‐C, Δ*tssD5*‐C, and Δ*tssD45*‐C were generated.

### 2.3 Phenotypic characteristics such as motility and toxoflavin synthesis between the *B. glumae* BGR1 and the *tssD* deletion mutants are not different

There were no differences in the growth rate between wild‐type *B. glumae* BGR1 and the single or quadruple *tssD* deletion mutants (Figure S3). Several virulence‐related phenotypic characters were tested. However, no apparent difference in swarming motility was observed in the mutant strains compared to that in wild‐type BGR1, that is, comparable swarming motility was observed in all strains, as assessed by the formation of dendritic patterns (Figure S4a). Furthermore, none of the strains had any defects in the biosynthesis of toxoflavin (Lee *et al*., 2016) (Figure S4b). Thus, there were no changes in the major phenotypic characteristics, including the major virulence factors of *B. glumae* BGR1, in *tssD* deletion mutants despite the loss of T6SS functions.

### 2.4 T6SS group_4 and T6SS group_5 contribute to virulence toward rice plants

**Figure 2:**
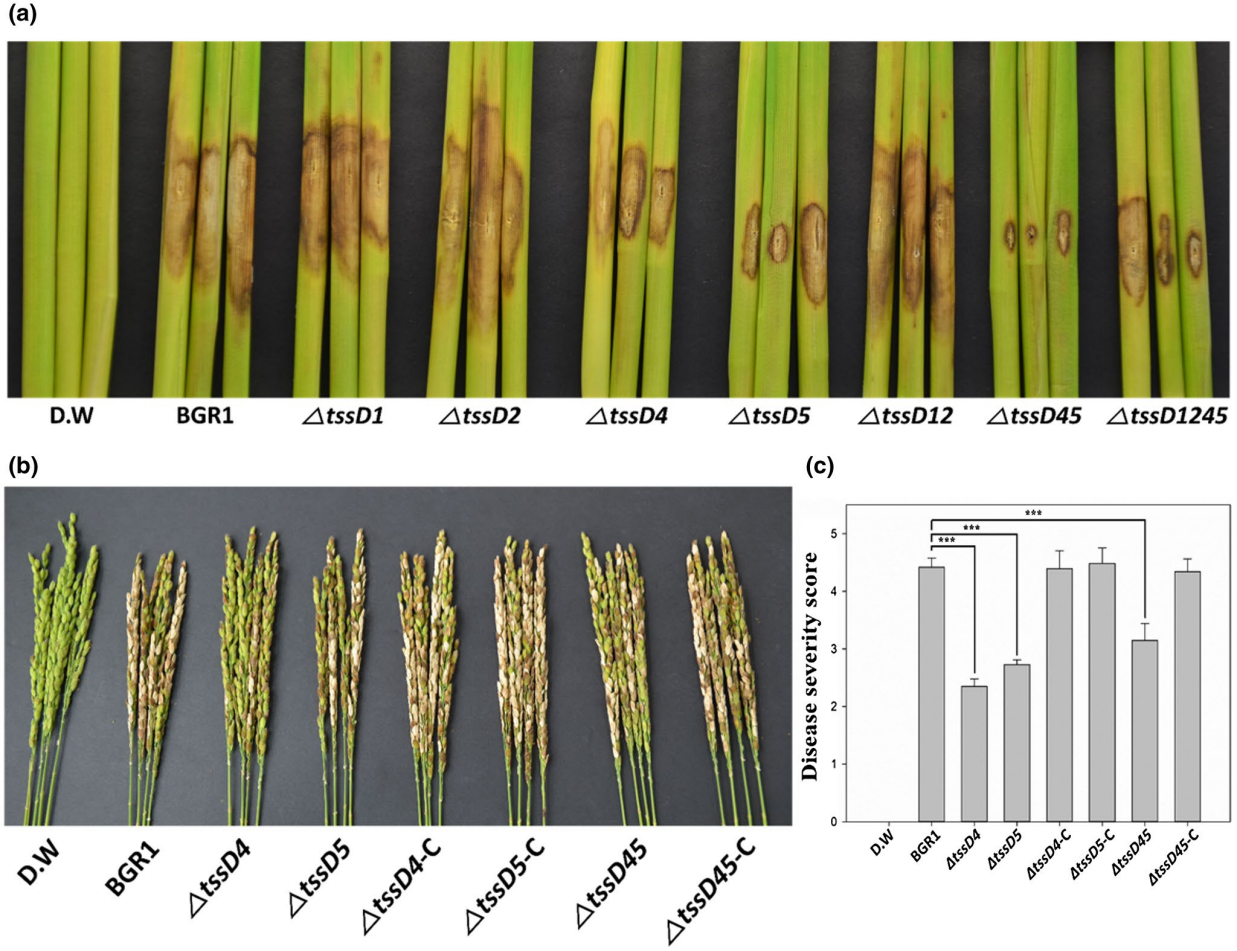
In vivo pathogenicity assay of the vegetative and reproductive stage. (a) T6SS deletion mutants were inoculated with 10^8^ cfu/ml to assess the virulence of the T6SS cluster in the xylem of rice stem (*Oryza sativa*) at the vegetative stage. The experiments were conducted by three replicates (*n* = 3). (b) Bacterial suspensions were inoculated into rice panicles at the reproductive stage to assess the virulence of Δ*tssD4,* Δ*tssD5*, Δ*tssD45*, Δ*tssD4*‐C, Δ*tssD5*‐C, and Δ*tssD45*‐C. Representative of four replicates. (c) Disease severity on the rice panicles was calculated on a scale of 0 to 5 after inoculating the bacterial suspension. The data are presented as the mean ± *SD* of four replicates (*n* = 4). Mean values followed by the same letters are not significantly different according to Tukey's HSD test (*p < .05, **p < .01, ***p < .001). Disease symptoms at 8 days post‐inoculation. Distilled water (D.W.) was used as the negative control

To determine whether the T6SS is involved in the eukaryotic cell‐targeting system of *B. glumae* BGR1, rice plants were infected by wild‐type *B. glumae* BGR1 and *tssD* mutants. Primary virulence assays were conducted towards rice plants to investigate the relationship between the T6SS and virulence in the vegetative stages of rice cultivated under greenhouse conditions. At 8 days post‐inoculation (dpi) of the tested *tssD* mutants (Δ*tssD1,* Δ*tssD2,* Δ*tssD4,* Δ*tssD5*, Δ*tssD12*, Δ*tssD45*, and Δ*tssD1245*), only Δ*tssD4*, Δ*tssD5*, Δ*tssD45*, and Δ*tssD1245* showed reduced virulence (Figure 2a). These results suggest that the T6SSs group_4 and group_5 were involved in bacterial virulence towards rice plants as the eukaryotic cell‐targeting system.

Next, the bacterial suspensions of Δ*tssD4*, Δ*tssD5*, and Δ*tssD45* were inoculated in the reproductive stage of rice plants to determine whether the T6SSs are the virulence factors in bacterial panicle blight caused by BGR1. By 8 dpi, most of the BGR1‐inoculated panicles had developed blight symptoms. The results of the virulence test for Δ*tssD4*, Δ*tssD5*, and Δ*tssD45* in rice panicles were consistent with those obtained in the virulence assay at the vegetative stage of rice. Specifically, disease severity (0–5) was 4.42 ± 0.13 in wild‐type BGR1, and decreased to 2.35 ± 0.11 in Δ*tssD4*, to 2.73 ± 0.07 in Δ*tssD5*, and to 3.15 ± 0.24 in Δ*tssD45*. Furthermore, the complemented strains, Δ*tssD4*‐C, Δ*tssD5*‐C, and Δ*tssD45*‐C, almost completely recovered their virulence, and the disease severity of these strains was 4.40 ± 0.25, 4.48 ± 0.22, and 4.34 ± 0.18, respectively (Figure 2b,c). BGR1 pBBR1MCS2, the negative control strain of the complementation strain, showed virulence towards rice plants similar to the wild‐type BGR1 (Figure S5a–c). Thus, the genes *tssD4* and *tssD5* contribute to virulence towards rice plants regardless of the growth stage of the plant. However, the result of no attenuated virulence by Δ*tssD45* showed no synergistic effect via *tssD4* and *tssD5*.

### 2.5 T6SS group_1 is involved in antibacterial competition ability

**Figure 3:**
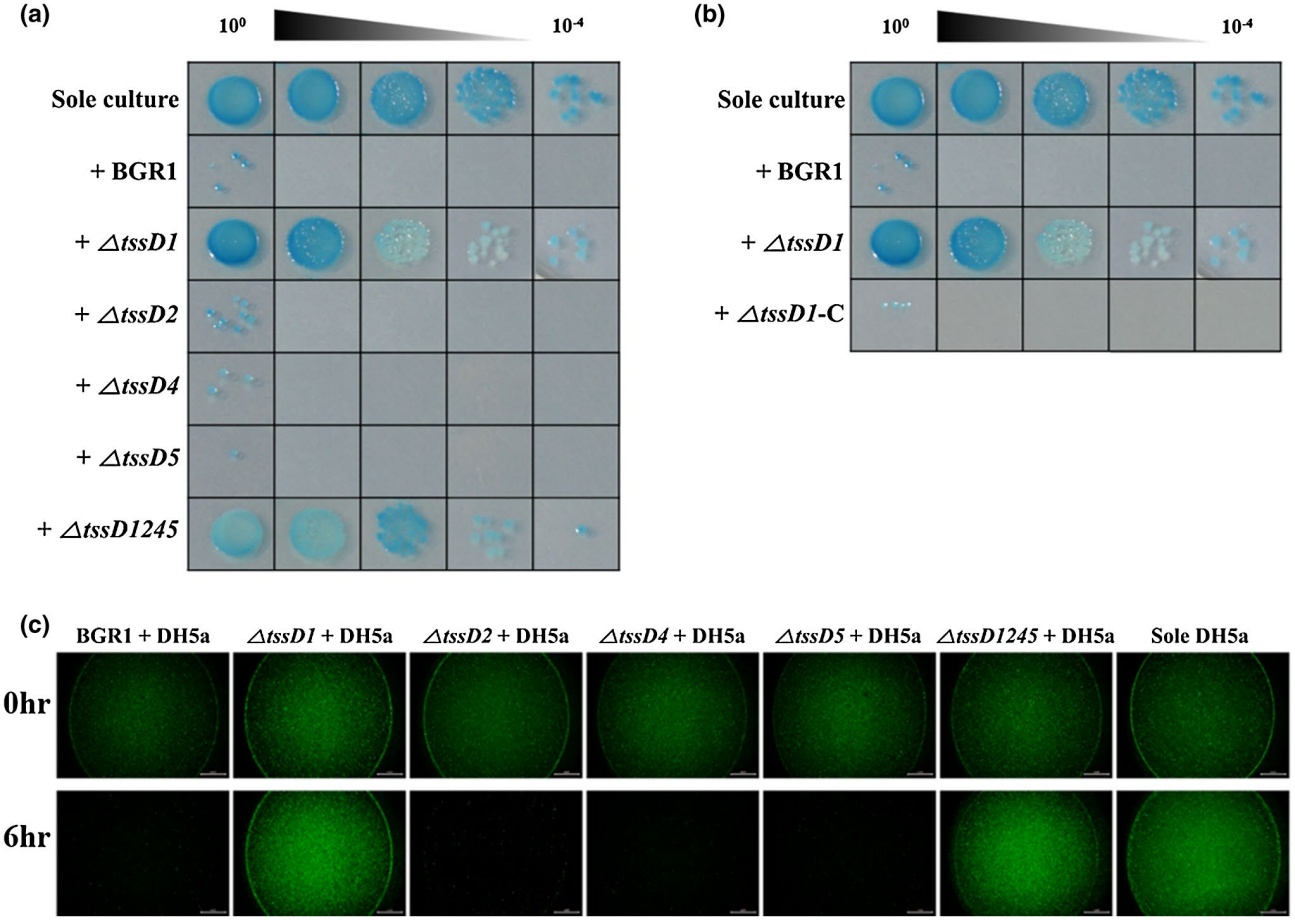
Interbacterial interaction between *Escherichia coli* DH5α and four *tssD* mutants of *Burkholderia glumae* BGR1. (a) and (b) Antibacterial effects of the four T6SS clusters in *B. glumae* BGR1. Survival of prey cell was strongly decreased by coculturing with wild‐type BGR1, Δ*tssD2*, Δ*tssD4*, and Δ*tssD5*. The survival of prey cells by coculture with Δ*tssD1* and Δ*tssD1245* was similar to that of pure culture of prey without predators (sole). The Δ*tssD1* complementation strains restored the wild‐type antibacterial activity. The experiments were conducted by three replicates (*n* = 3). (c) Observation of living prey cells expressing green fluorescent protein (GFP) by spotting the prey and predator mixture. Except in the coculture of Δ*tssD1* and Δ*tssD1245*, the intensity of GFP expressed in living prey cells was found to disappear after 6 hr. The experiments were conducted using three replicates (*n* = 3). This is representative of the results from independent experiments with three replicates showing the same pattern

The antibacterial competition ability was tested using apramycin‐resistant *Escherichia coli* cells as prey and cocultured *B. glumae* BGR1 as predators. The single *E. coli* cell cultures proliferated to approximately 9.33 × 10^7^ cfu/ml, whereas *E. coli* cocultured with an equal number of wild‐type BGR1 cells at a 1:1 ratio for 6 hr showed a decrease to 2.67 × 10^3^ cfu/ml. As a result, the surviving number of *E. coli* cells grown in pure culture was more than 10^4^ times higher compared to those cocultured with wild‐type BGR1. To investigate whether the T6SS of *B. glumae* BGR1 is involved in this antibacterial effect, the numbers of *E. coli* cells surviving coculture with Δ*tssD1245* were compared with those of *E. coli* cells cultured alone. The number of *E. coli* surviving coculture with Δ*tssD1245,* which proliferated to approximately 3.25 × 10^7^ cfu/ml, was similar to that of *E. coli* cultured alone. Next, antibacterial competition assays with *tssD1*, *tssD2*, *tssD4*, and *tssD5* single mutants as predators were conducted to determine which T6SS clusters were associated with the antibacterial effects (Figure 3a). Only the effect of Δ*tssD1* was found to be comparable with that of Δ*tssD1245*. The numbers of *E. coli* cells surviving coculture with Δ*tssD1* proliferated to approximately 2.67 × 10^7^ cfu/ml. The complemented strain Δ*tssD1*‐C, with recovered function of *tssD1*, showed antibacterial activity almost completely restored to wild‐type levels, as reflected by a sharp decrease in the surviving number of *E. coli* prey cells (Figure 3b). The BGR1 pBBR1MCS2 strain, as the negative control of the complementation strain, showed an antibacterial effect similar to wild‐type BGR1 (Figure S5d). The antibacterial effect of Δ*tssD1* and Δ*tssD1245* was also observed using green fluorescent protein (GFP)‐labelled *E. coli* as prey (Figure 3c). Therefore, only the *tssD1* gene contributed to the antibacterial effect in in vitro interbacterial competition assays.

### 2.6 The presence of prey increases the gene expression levels of *tssD1* but not *tssD2*, *tssD4*, and *tssD5*

**Figure 4:**
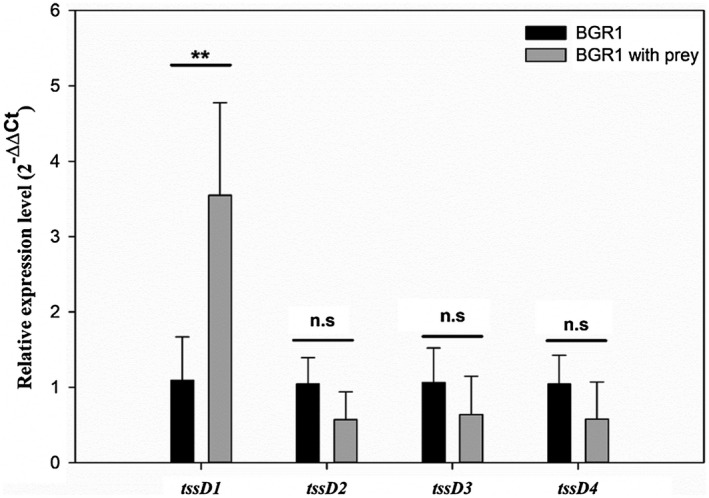
Analysis of relative gene expression level of *tssD1, tssD2*, *tssD4*, and *tssD5* with or without prey. The relative gene expression levels of the four *tssD* genes of *Burkholderia glumae* BGR1 in the absence or presence of *Escherichia coli* as prey are presented. Only *tssD1* gene expression was increased in the presence of *E. coli* as prey. The experiments were conducted by four replicates (*n* = 4). Mean values followed by the same letters are not significantly different according to Tukey's HSD test (ns, no statistical significance, **p < .01)

Quantitative reverse transcription PCR (RT‐qPCR) was used to assess the expression levels of the four *tssD* genes in the presence of prey. In the presence of *E. coli* as prey, the relative gene expression level of only *tssD1* was increased whereas the relative gene expression levels of the *tssD2*, *tssD4*, and *tssD5* genes showed little difference regardless of the presence of prey (Figure 4).

### 2.7 The endophytic bacterial community composition in noninfected rice, rice infected with *B. glumae* BGR1, and rice infected with Δ*tssD1* differ because of the antibacterial activity of T6SS group_1

**Figure 5:**
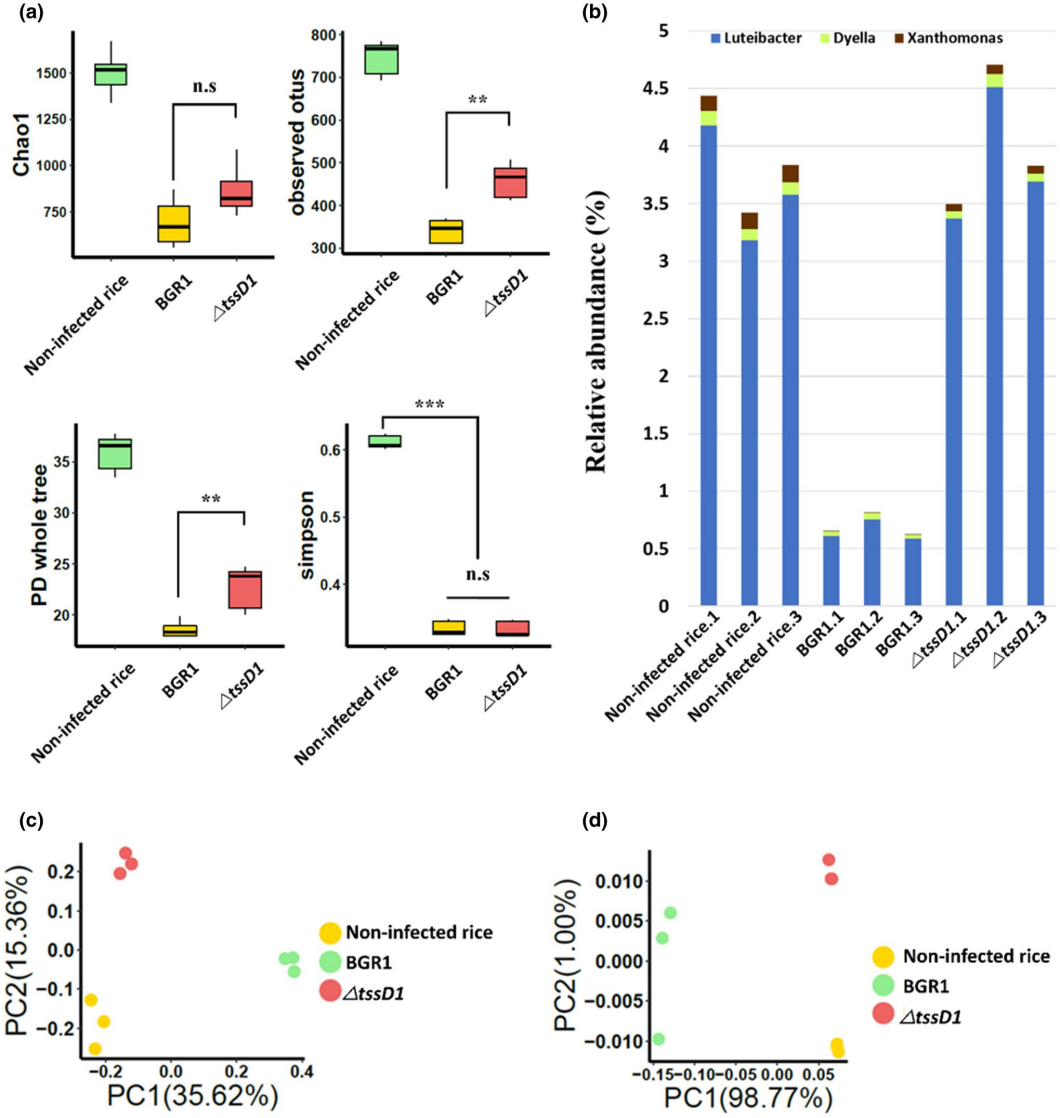
Alpha diversity and beta diversity in noninfected rice, rice infected by *Burkholderia glumae* BGR1 and the Δ*tssD1* mutant. (a) Species richness (Chao1), observed operational taxonimoc units (OTUs), phylogenetic diversity (PD), and Simpson evenness was estimated. Alpha diversity was higher in the noninfected rice samples than in the other two groups. Apart from the Simpson index, the three indices showed that rice infected by Δ*tssD1* samples showed greater diversity than that in rice infected by BGR1. The box‐and‐whiskers plot represents the range of the upper quartile and lower quartile, and the median value. (b) *Luteibacter*, *Dyella*, and *Xanthomonas* were the most dominant in noninfected rice and in rice infected rice with Δ*tssD1* in the relative abundance of the top 20 bacteria at genus level. Principal coordinate analysis plots of (c) unweighted UniFrac and (d) weighted UniFrac distance of bacterial community composition. There was a significant difference in bacterial composition by the group (*p* = .001, *p* = .006, respectively, ANOSIM) in both weighted and unweighted UniFrac distances

Metagenomic analysis of endophytic bacterial communities was performed among three groups: noninfected rice, rice infected with BGR1, and rice infected with Δ*tssD1*. Alpha diversity was used to measure the diversity of each sample by calculating a value for each sample. Alpha diversity was quantified based on Chao1, the observed operational taxonomic units (OTUs), the phylogenetic diversity (PD) whole tree, and the Simpson index (Figure 5a). Alpha diversity between rice infected with BGR1 and rice infected with Δ*tssD1* showed that the observed OTUs (*P*_observed OTUs_ = 0.003) and PD whole tree (*P*_PD whole tree_ = 0.008) were significantly decreased in rice infected with BGR1, even though statistical testing using Chao1 (*P*_Chao1_ = 0.052) and the Simpson index (*P*_Simpson_ = 0.408) showed no difference (Figure 5a). All alpha diversity indices of noninfected rice were higher than the other groups. The relative abundance of the top 20 genera showed that the genus *Pantoea* dominated the noninfected rice sample, followed by the genus *Burkholderia*. In contrast, the most abundant genera in rice infected with BGR1 and Δ*tssD1* were *Burkholderia* followed by *Achromobacter* (Figure S6). Bacteria in the genera *Luteibacter*, *Dyella*, and *Xanthomonas* were relatively more abundant in the samples of noninfected rice and rice infected with Δ*tssD1* than in rice infected with BGR1 (Figure 5b).

**Figure 6:**
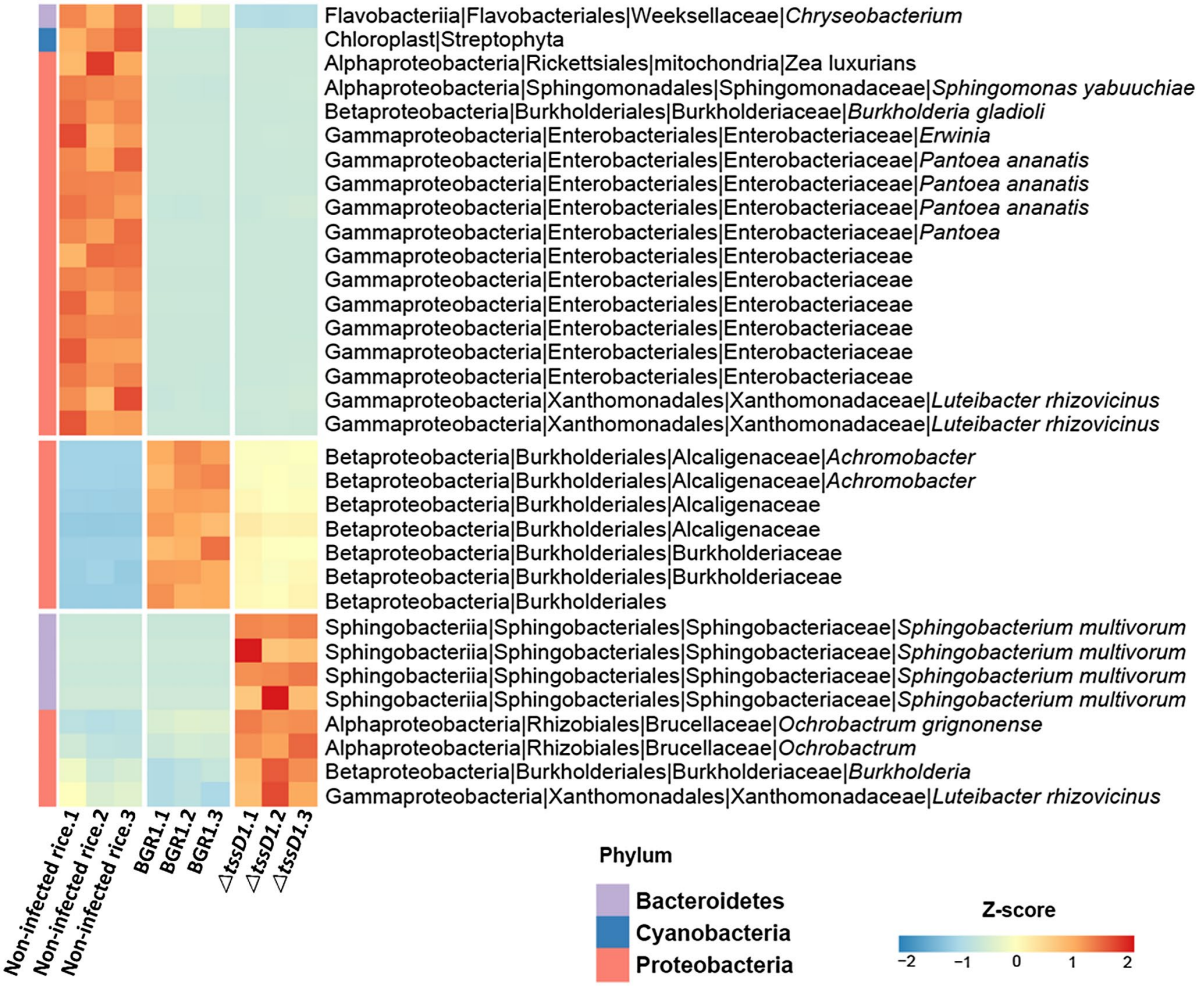
Identifying taxa with the most differences abundance from LEfSe analysis. LefSe analysis was performed with *p* < .05, LDA score > 2.5 with the relative abundance of bacterial OTUs. Blue represents lower abundance and red represents higher abundance. Relative abundances were normalized to *z*‐score to indicate differences between samples. The side bar on the left of the heatmap indicates the bacterial phyla

Principal coordinate analysis (PCoA) plots showed the difference in the communities of the noninfected rice, rice infected with BGR1, and rice infected with Δ*tssD1* based on the distance metrics obtained from UniFrac analysis (Figure 5c,d). Endophytic bacterial communities in the noninfected rice, rice infected by BGR1, and rice infected by Δ*tssD1* were separated clearly by a principal coordinate plot of distance based on the analysis of a weighted and unweighted UniFrac (*p* = .001, .006, respectively) (Figure 5c,d). Among these three groups, linear discriminant analysis (LDA) effect size (LEfSe) was used to find the strongest effects for group differentiation using imbalanced OTUs. Heatmap abundance showed differential abundance and frequency of the total bacterial groups (*p* < .05, LDA score > 2.5) among the three groups (Figure 6). PCoA plots and heatmap abundance (*p* < .05, LDA score > 2.5) showed that the three groups constituted their own communities and presence or absence of antibacterial effects led to differences in the community of rice infected with BGR1 and Δ*tssD1* at 8 dpi (Figures 5 and 6). At the order level, *Enterobacteriales*, *Flavobacteriales*, *Bacillales*, and *Rickettsiales* were enriched in noninfected rice, whereas *Rhizobiales* and *Sphingobacteriales* were enriched in rice infected by Δ*tssD1*. In rice infected by BGR1, only *Burkholderiales* was observed at the order level. At the family level, six bacterial groups, *Enterobacteriaceae*, *Sphingomonadaceae*, *Flavobacteriaceae, Rickettsiaceae*, *Rhizobiaceae*, and *Staphylococcaceae*, were enriched in noninfected rice; five bacterial groups, *Sphingobacteriaceae*, *Brucellaceae*, *Oxalobacteraceae*, *Burkholderiaceae*, and *Comamonadaceae*, were enriched in rice infected by Δ*tssD1*; and only one bacterial group, *Alcaligenaceae*, was enriched in rice infected rice by BGR1. In particular, the order *Flavobacteriales* in noninfected rice and the order *Sphingobacteriales* in rice infected by Δ*tssD1* were abundant in the phylum *Bacteroidetes*, whereas at the phylum level, Proteobacteria were enriched in rice infected by BGR1.

## 3 DISCUSSION

Type VI secretion systems (T6SSs) have been discovered in a variety of gram‐negative bacteria. The T6SS is known to have two distinct features: antibacterial effects as bacterial interaction and bacterial virulence as eukaryotic interaction. In many *Burkholderia* species having at least one T6SS, the T6SS has evolved into the most complex secretion pathway and has an essential role in their interaction with the environment (Nguyen *et al*., 2018). Based on the evolutionary analysis of T6SSs in *Burkholderia* species, T6SS group_1 of *B. glumae* BGR1 was clustered in bacterial interaction with antibacterial effect, whereas T6SS group_4 and group_5 were clustered in eukaryotic interaction with bacterial virulence; the function of T6SS group_2 could not be predicted through evolutionary analysis. To identify the T6SSs of *B. glumae* BGR1, the approach of evolutionary relationships is useful for inferring the function and role of a specific relationship. However, additional information is needed to determine whether the evolutionary relationship of a T6SS suggests general predictable factors for the function of the T6SS in eukaryotic and bacterial cell interactions.

*B. glumae* BGR1 has four T6SS gene clusters, namely two complete T6SS gene clusters (T6SS group_2, 4) and two incomplete T6SS gene clusters (T6SS group_1, 5), in its genome. The T6SS group_1 gene cluster is incomplete due to lack of the *vgrG* gene. However, some *vgrG* genes are located outside the T6SS gene cluster and are used as major components of the T6SS (Zong *et al*., 2019). The T6SS group_5 gene cluster is also incomplete due to three major components being missing. Although *Yersinia pestis* CO92 also has five incomplete T6SS gene clusters among six T6SS gene clusters, the five incomplete T6SS clusters seem to be involved in host cell cytotoxicity based on the results of infection attenuation with the incomplete T6SS deletion mutants (Andersson *et al*., 2017; Yang *et al*., 2018). Thus, an incomplete T6SS cluster, such as T6SS group_1 and 5, could be functional.

Based on our evolutionary analysis, we hypothesized that the four T6SS gene clusters of *B. glumae* BGR1 are evolutionarily differentiated and play distinct roles in the environment. The construction of deletion mutants to knock out the function of the T6SS needle were attempted by targeting *tssD* genes, which are present in each of the four T6SS clusters of *B. glumae* BGR1. The TssD protein, which forms an inner tube of stacked hexamers with helical symmetry on the assembly baseplate to transport effectors, is essential to form the T6SS needle to puncture adjacent cells (Pell *et al*., 2009; Cascales and Cambillau, 2012). The T6SS needle, with the VgrG‐PAAR spike complex at its end, comprises an inner tube and a TssB‐TssC ring surrounding the inner tube. Interestingly, Hcp (TssD) and VgrG proteins are mutually dependent when the surface is assembled (Pukatzki *et al*., 2007; Zheng and Leung, 2007; Hachani *et al*., 2011). Consequently, *tssD* deletion leads to loss of function of the T6SS in *B. glumae* BGR1 because the TssD protein is required to build an intact T6SS.

The T6SS is involved in bacterial virulence as a eukaryotic cell targeting system for plants or animals. The T6SSs as a eukaryotic cell targeting system of animals have been reported a to be essential elements for the virulence of bacterial pathogens (Keynan and Rubinstein, 2007; Suarez *et al*., 2010; Russell *et al*., 2013). Furthermore, the T6SSs, as a eukaryotic cell targeting system of plants, have been identified important for bacterial virulence based on an observable decrease of bacterial virulence in plants in the absence of the T6SS (Zhang *et al*., 2014; Shyntum *et al*., 2015). An in vivo pathogenicity assay using *tssD* deletion mutants was performed in rice stems at the vegetative stage to determine whether the T6SSs of *B. glumae* BGR1 are required for bacterial virulence. The attenuated virulence of Δ*tssD4* and Δ*tssD5* in the in vivo pathogenicity assay of stems suggests that T6SS group_4, including the *tssD4* gene, and T6SS group_5, including the *tssD5* gene, are related to bacterial virulence and pathogenicity in rice plants. The results of rice panicle blight disease by an in vivo pathogenicity assay at the reproductive stage were consistent with those from the stems at the vegetative stage, confirming that *tssD4* and *tssD5* are required for the full virulence of *B. glumae* BGR1 in rice plants. However, the results of no attenuated virulence by Δ*tssD45*, compared to Δ*tssD4* and Δ*tssD5*, indicate the absence of a synergistic effect in causing bacterial panicle blight by the genes *tssD4* and *tssD5* (Figure 2b,c). Thus, T6SS group_4 and T6SS group_5 contribute to bacterial virulence as a completely independent eukaryotic targeting system.

The T6SS, involved in the eukaryotic targeting system, is activated when certain conditions are met on entering the host. For example, T6SS‐1 of *B. pseudomallei*, which is involved in virulence in mice and hamsters, is not expressed during culture in rich media but is expressed following uptake by the host cell (Shalom *et al*., 2007; Burtnick *et al*., 2011; Chen *et al*., 2011). Only T6SS group_5 of *B. glumae* was expressed and activated during infection of rice in Table S1 (Kim *et al*., 2014). However, the exact mechanism for the involvement of T6SS group_4 and T6SS group_5 in the infection process and for the activation of T6SSs remains unclear. For a comprehensive understanding of the T6SS activation mechanisms, bioinformatics and comparative secretome approaches need to be employed in future studies.

In addition to engaging in bacterial virulence toward host organisms, the T6SS is involved in bacterial competition via antibacterial effects by T6SS‐dependent antibacterial effectors like peptidoglycan hydrolases, phospholipases, nucleases, and NAD(P)^+^‐glycohydrolases (Koskiniemi *et al*., 2013; Russell *et al*., 2013; Whitney *et al*., 2013; Tang *et al*., 2018). Bacterial competition between species appears to be defined as interactions for occupying a predominant position in the bacterial community. In general, bacterial competition occurs within the framework of growth suppression or limitation. Our in vitro interbacterial competition assay showed that only T6SS group_1 plays an important role in bacterial competition with *E. coli* through suppression or limitation of bacterial growth. The similar survival rate of prey cells between coculture with Δ*tssD1* and Δ*tssD1245* indicated that the other T6SS groups have no synergistic effects with T6SS group_1 (Figure 2a). Although we conducted a bacterial competition assay under limited conditions using *E. coli* or GFP‐labelled *E. coli* as prey, it may be a suitable approach to investigate interbacterial interactions of T6SS group_1 in *B. glumae* BGR1. T6SS‐related recognition of *Proteus mirabilis* requires cell‐to‐cell contact for the usage of T6SSs, which discriminate the strains and orchestrate the neighbouring cells (Saak and Gibbs, 2016; Gallique *et al*., 2017), Thus, we performed a RT‐qPCR analysis to explore the expression pattern of four *tssD* genes of *B. glumae* BGR1 in the presence of *E. coli* as prey. When *B. glumae* BGR1 was present with *E. coli* as prey, the expression level of only the *tssD1* gene was increased. Consequently, the high expression level of *tssD1* suggests that *B. glumae* BGR1 recognizes external stimuli such as the presence of *E. coli* as prey, and activates the T6SS group_1, which is involved in the antibacterial effect.

In order to investigate the antibacterial effect of T6SS group_1 involved in bacterial competition in host plant by using infection of Δ*tssD1*, metagenomic analysis was performed by focusing on the different bacterial communities in rice plants infected with the Δ*tssD1* mutant compared to the wild‐type BGR1. Metagenomic analysis of the endophytic bacterial community showed upon infection by the plant‐pathogenic *B. glumae* BGR1 in rice plants, the existing bacterial population in the plant collapsed, and *B. glumae* BGR1 formed the dominant population causing plant disease. The bacterial population in noninfected rice plants had a much higher diversity compared to the bacterial population in rice infected by *B. glumae* BGR1 and by Δ*tssD1*. There was no significant difference in the Simpson index, reflecting the relative abundance and the number of species between the groups of rice infected by *B. glumae* BGR1 and by Δ*tssD1* due to the high relative abundance of above 80% of genus *Burkholderia*. However, the differences in the unique OTUs and PD whole tree between the rice infected by *B. glumae* BGR1 and Δ*tssD1* is a result of deletion of the *tssD1* gene from T6SS group_1. Relative abundance at the genus level also showed that the genera *Luteibacter* and *Dyella*, belonging to Gammaproteobacteria, were more abundant in rice infected by Δ*tssD1* than by BGR1. In particular, the genus *Luteibacter* contains *L. rhizovicinus*, a bacterial species that promotes plant growth and development, and contributes to protection against pathogenic bacteria (Innerebner *et al*., 2011; Guglielmetti *et al*., 2013). Consequentially, the T6SS group_1 was shown to play a role in the antibacterial effect by targeting endophytic plant‐associated bacteria within rice plants and created an easily accessible environment for other pathogenic bacteria by controlling plant growth‐promoting bacteria. However, these results have limitations in explaining the differences in the overall bacterial population between the groups of rice infected with *B. glumae* BGR1 and Δ*tssD1*.

Although the disease severity in rice infected with *B. glumae* BGR1 and Δ*tssD1* was similar, LEfSe analysis showed differences in the bacterial community among the three groups, noninfected rice, rice infected by *B. glumae* BGR1, and rice infected by Δ*tssD1*. In particular, endophytic plant‐associated bacteria in the orders *Enterobacteriales*, *Flavobacteriales*, *Sphingomonadales*, *Bacillales*, *Rickettsiales*, *Sphingobacteriales*, and *Rhizobiales* were dominant in noninfected rice and in rice infected by Δ*tssD1* (Ivanova *et al*., 2000; Delmotte *et al*., 2009). In contrast, in rice infected by *B. glumae* BGR1, the order *Burkholderiales*, which contains several pathogenic bacterial species, prevailed (Hauser *et al*., 2011; Voronina *et al*., 2015). Thus, T6SS group_1 of *B. glumae* BGR1 is not involved in bacterial virulence, but has the function of exerting antibacterial effects by recognizing certain surrounding endophytic plant‐associated bacteria like *Luteibacter* and *Dyella* as prey; the growth inhibition of endophytic plant‐associated bacteria by antibacterial effects is important for bacterial competition in rice plants. In particular, bacterial competition is more important to form dominant populations of plant pathogens like *B. glumae* BGR1 during their initial colonization in plants to cause plant disease.

These results allow us to infer the distinct role of T6SSs in the infection process of *B. glumae* BGR1. Moreover, investigation of *tssD* gene expression profiles provided insights into the role of these genes in vitro and in planta. *B. glumae* BGR1 must reach a sufficient cell population to cause disease. *B. glumae* BGR1 recognizes the presence of surrounding bacteria and uses T6SS group_1 to gain an advantage in the ecological niche by competing with adjacent bacteria inside rice plants. In the next step, *B. glumae* BGR1, predominant in rice, demonstrates interaction between bacteria and plants through bacterial virulence by targeting the eukaryotic cells of the rice plant either directly or indirectly through T6SS group_4 and T6SS group_5.

In the current study, we provide the first functional description of different T6SSs in the phytopathogenic *B. glumae* and provide evidence for their roles in interbacterial interaction and virulence towards rice plants. The T6SS group_1 confers bacterial competition ability in rice plants, whereas T6SS group_4 and T6SS group_5 are involved in the virulence toward host plants. Our results indicate that the T6SSs of *B. glumae* BGR1 are involved in the plant–bacteria interaction and in the interbacteria interaction, and play a functionally distinct role as efficient and indiscriminate weapons in causing plant disease.

## 4 EXPERIMENTAL PROCEDURES

### 4.1 Analysis of type VI secretion systems in the genome of *Burkholderia* species using the maximum‐likelihood method

The available T6SS clusters, including Clusters of Orthologous Groups of proteins (COG) of T6SS major components, which are COG3515; TssA, COG3516; TssB, COG3517; TssC, COG3157; TssD (Hcp), COG3518; TssE, COG3519; TssF, COG3520; TssG, COG0542; TssH, COG3501; TssI (VgrG), COG3521; TssJ, COG3522; TssK, COG3455; TssL; and COG3523; TssM in some bacterial genomes, were acquired from the SecreT6 database (http://db‐mml.sjtu.edu.cn/SecReT6/) (Li *et al*., 2015) and their nucleotide sequences were extracted from the National Center for Biotechnology Information (NCBI). Next, these T6SS component genes were detected in the genome of 10 *Burkholderia* species, which includes *B. glumae* BGR1, using the T6SS‐BLASTP program of SecreT6. All T6SS component genes in *Burkholderia* species were confirmed in detail. In particular, the COGs of 13 conserved T6SSs were found in four different clusters on the genome of *B. glumae* BGR1 among 10 *Burkholderia* species and were assigned to each gene encoding similar proteins in the four clusters (Figure 1). Several types of T6SS cluster, including at least 10 major components, were used to elucidate the relationship among all detected T6SS clusters of *Burkholderia* species. The protein sequences of all T6SS clusters were aligned using the ClustalW algorithm in MEGA6. The relationships of *Burkholderia* T6SS were constructed based on the maximum‐likelihood method and the Jones‐Taylor‐Thornton matrix‐based model with 1,000 bootstrap replicates (Jones *et al*., 1992). The relationship and evolutionary rate differences among sites (five categories [+G, parameter = 4.9]) were designated with a discrete gamma distribution.

### 4.2 In vivo pathogenicity assay at vegetative stage and reproductive stage

To examine the eukaryotic‐targeting system of *B. glumae* BGR1 in rice plants at the vegetative stage and reproductive stage, cultured bacterial cells at the mid‐logarithmic phase were harvested by centrifugation, washed, and resuspended with distilled water. The optical density of bacterial suspensions was adjusted to OD_600 nm_ = 0.8. The rice plants used in this experiment were grown under greenhouse conditions (average 30°C in the day and 25°C at night). Next, the stems of rice plants in the vegetative stage were inoculated with bacterial suspensions using a syringe and grown for 8 days. The disease severity was observed at the inoculated area. Suspensions for each bacterial stain were prepared and adjusted to OD_600 nm_ = 0.5 to confirm the bacterial panicle blight disease at the reproductive stage of rice. At the flowering stage, the rice panicles were inoculated by dipping them into 50 ml of bacterial suspensions for 1 min. At 8 dpi, the disease severity in rice panicles was evaluated using the following scale: 0, healthy panicle; 1, 0%–20% diseased panicle; 2, 21%–40% diseased panicle; 3, 41%–60% diseased panicle; 4, 61%–80% diseased panicle; 5, 81%–100% diseased panicle. Disease severity was calculated using the following formula: disease severity = Σ(number of samples per rating × rating value)/total number of panicles. BGR1 pBBR1MCS2, which contains an empty vector, was used as a negative control of the complementation strain in the in vivo pathogenicity assay.

### 4.3 In vitro interbacterial competition assay

*B. glumae* strains (Table S1) were cocultured with *E. coli* DH5α, used as prey cells. The *E. coli* DH5α cells harboured the pCRISPomyces‐2 plasmid, allowing survival in apramycin medium and alpha complementation for β‐galactosidase. The *B. glumae* strains were co‐incubated with *E. coli* at a 1:1 ratio in the form of patches on Luria Bertani (LB) plates for 6 hr at 37°C. Following incubation, the patches were recovered and resuspended in LB broth. To assess the survival of the *E. coli* prey, the resuspended mixtures were serially diluted from 0 to 10^−7^ and spotted on LB plates containing apramycin (100 μg/ml) and 5‐bromo‐4‐chloro‐indolyl‐β‐d‐galactopyranoside (X‐Gal, 40 μg/ml). The number of blue colonies on the plate indicated the number of surviving *E. coli*. BGR1 pBBR1MCS2, which contains an empty vector, was used as a negative control of the complementation strain in the in vitro interbacterial competition assay. In addition, an interbacterial competition assay with GFP was performed with the same method using GFP‐labelled *E. coli* DH5α. The *gfp* gene was amplified from the commercial plasmid pUBN‐GFP‐DEST (Addgene) using the primer pair GFP_HindIII_F and GFP_BamHI_R (Table S3). The amplified *gfp* fragment and pBBR1P2 plasmid were ligated. Recombinant plasmids were transformed into *E. coli* DH5α. The density of GFP‐labelled *E. coli* was observed at 0 and 6 hr of bacterial competition under an Optinity KB‐600F fluorescent microscope with a CoolLED pE300‐WHITE illumination (CoolLED). Images were acquired with the KCS3‐23S CMOS sensor camera (KoreaLabTech).

### 4.4 Isolation of bacteria from rice plants and preparation of metagenomic DNA

Stems of rice plant at the vegetative stage were inoculated with each bacterial suspension as explained above (adjusted to OD_600 nm_ = 0.8). At 8 dpi, bacteria were isolated from the noninfected and infected stems of rice. The noninfected or infected rice stem segments at 5 cm from the upper and lower parts of the inoculation site were gathered. The surface of each 10 cm rice stem segment was sterilized to eliminate the bacterial cells attached to the surface of the plant tissue by wiping it down with 70% ethanol. Next, about 6 g of six rice stem segments were squeezed tightly in a 15‐ml conical tube, and centrifuged at 4°C and 1,800 × g for 7 min directly to harvest a pellet of the bacteria inside the stem segments. The bacterial pellet acquired after centrifugation was immediately used to extract DNA following the classic cetyltrimethyl ammonium bromide‐based protocol with modifications (Murray and Thompson, 1980). The extracted DNA was amplified to obtain paired‐end sequences of the 16S rRNA variable regions using Herculase II fusion DNA polymerase Nextera XT Index Kit v. 2 on the Illumina MiSeq platform at Macrogen (Seoul, South Korea). The following primer pairs were used for amplification: (F), 5′‐TCGTCGGCAGCGTCAGATGTGTATAAGAGACAGCCTACGGGN‐GGCWGCAG‐3′; (R), 5′‐GTCTCGTGGGCTCGGAGATGTGTATAAGAGACAGGACTACHVGGGTATCTAATCC‐3′.

### 4.5 Bacterial community analysis

The paired‐end sequences with a *p* value of 0.3, obtained from Illumina MiSeq, were merged using meren/illumina‐utils (https://github.com/meren/illumina‐utils) (Eren *et al*., 2013). The merged sequences were analysed using the QIIME (Quantitative Insights into Microbial Ecology) pipeline v. 1.9.1 (Caporaso *et al*., 2010b). The total merged sequences were clustered into de novo OTUs with a sequence identity of 97% using UCLUST (Edgar, 2010). The representative sequences were designated using the most abundant sequences within each OTU. The representative sequences were aligned using PyNAST aligner (Caporaso *et al*., 2010a), and FastTree (Price *et al*., 2009) was used to construct a phylogenetic tree. The representative sequences of OTUs were assigned to a Greengenes database (DeSantis *et al*., 2006) using an RDP (Ribosomal Database Project Ⅱ (http://rdp.cme.msu.edu)) Classifier (Wang *et al*., 2007) with a threshold confidence of 50%. OTUs assigned as chloroplasts and mitochondria were excluded from further analysis. As *B. glumae* was not included in the Greengene database, BLASTn was performed using the representative sequences against the NCBI ref‐seq database to identify sequences with more than 97% sequence identity as *B. glumae*. Beta diversity was estimated using the unweighted and weighted UniFrac distance matrices with a phylogenetic tree. A PCoA was conducted to identify the bacterial community differences among samples using UniFrac distance matrices. Alpha diversity was estimated with Chao1, PD whole tree, observed OTUs, and Simpson indices using rarefied OTU tables (96,526 sequences per sample).

### 4.6 Statistical analysis

All experiments except bacterial community analysis were conducted twice with at least three replicates. Bacterial community analysis was conducted three times with six replicates. Analysis of variance was conducted using the generalized linear model procedure and means were compared using the least significant difference test at *p* < .05 according to Tukey's HSD test. To identify the significant differences in bacterial features among the three groups, LEfSe was performed. A threshold of LDA scores of 2.0, and α = .05 for the Kruskal–Wallis test were applied to estimate the effect size of each of the differently abundant OTU features (Segata *et al*., 2011).

## Supporting information


**FIGURE S1** Relationship of *Burkholderia* type VI secretion systems. The relationship of *Burkholderia* type VI secretion systems was generated using 13 T6SS core components in the T6SS clusters of *Burkholderia* species. The relationship was inferred using the maximum likelihood method and Jones‐Taylor‐Thornton matrix‐based model. The percentages of replicate trees in which the associated taxa were clustered together in the bootstrap test (1,000 replicates) are shown next to the branches. A discrete gamma distribution was used to model the evolutionary rate differences among sites (five categories [+*G*, parameter = 4.9003]). Evolutionary analyses were conducted in MEGA X. The eukaryotic targeting system is marked in red and the bacterial targeting system is marked in blue

**FIGURE S2** Confirmation of *tssD* mutants using PCR. (a) Deletion mutants were generated and PCR was conducted using primers targeting the 5′‐upstream and 3′‐downstream regions of each *tssD* region to verify the mutant strains. (b) Agarose gel electrophoresis of the PCR products was performed to distinguish the wild‐type and mutant strains based on different fragment lengths. The PCR product size for each mutant strain was smaller than that obtained from wild‐type BGR1

**FIGURE S3** Growth curve of *Burkholderia glumae* BGR1 and *tssD* mutants. Wild‐type BGR1, *tssD* single to quadruple deletion mutants, and *tssD* complementation strains were grown overnight at 37 °C. Overnight bacterial cultures were diluted into fresh Luria broth and then incubated at 37 °C. Bacterial growth was monitored using samples withdrawn every 2 hr and measuring the optical density (600 nm). Compared to the growth pattern of the wild type, no difference was observed in the growth pattern of the mutants. Points indicate the mean values and error bars indicate standard deviations (*n* = 4)

**FIGURE S4** Phenotype assay of wild‐type *Burkholderia glumae* BGR1 and the *tssD* mutants. (a) Swarming motility by wild‐type BGR1 and the mutants Δ*tssD1*, Δ*tssD2*, Δ*tssD4*, Δ*tssD5*, and Δ*tssD1245*. The swarming motility assay was performed on 0.5% agar plates. The image is representative of three independent replicates (*n* = 3). (b) Biosynthesis of toxoflavin by wild‐type BGR1 and mutants Δ*tssD1*, Δ*tssD2*, Δ*tssD4*, Δ*tssD5*, and Δ*tssD1245*. Thin‐layer chromatography analysis was used to detect toxoflavin from the bacterial culture supernatant. The produced toxoflavin was examined visually in daylight and under UV at 365 nm. This is representative of the results from independent experiments with three replicates showing the same pattern

**FIGURE S5** Comparison of in vivo pathogenicity assay and antibacterial effect between wild‐type BGR1 and BGR1 pBBR1MCS2. (a) Wild‐type BGR1 and BGR1 containing empty vector were inoculated with 10^8^ cfu/ml to assess the virulence in rice stem at the vegetative stage. (b) Bacterial suspension of wild‐type BGR1 and BGR1 pBBR1MCS2, which contains empty vector of pBBR1MCS2, were inoculated into rice panicles (*Oryza sativa*) at the reproductive stage to comparison of the virulence. (c) Disease severity on the rice panicles was calculated on a scale of 0 to 5 after inoculating the bacterial suspension. The data are presented as the mean ± *SD* of three replicates (*n* = 3). Mean values followed by the same letters are not significantly different according to Tukey’s HSD test (ns, no statistical significance, *p < .05, **p < .01, ***p < .001). At the reproductive and vegetative stage, the disease severity of rice infected with wild‐type BGR1 was not different from that of rice infected with BGR1 pBBR1MCS2. Disease symptoms at 8 days post inoculation. Distilled water was used as the negative control. (d) Antibacterial effects in wild‐type BGR1 and BGR1 pBBR1MCS2. Survival of prey cell was decreased by coculturing with wild‐type BGR1 and BGR1 pBBR1MCS2. Prey cell survival by coculturing with BGR1 pBBR1MCS2 was similar to prey cell survival by coculturing with wild‐type BGR1. This is representative of the results from independent experiments with three replicates showing the same pattern. BGR1 pBBR1MCS2 was used as the negative control of complementation strains. The data were conducted with three replicates

**FIGURE S6** Relative abundance of the top 20 genera at the genus level. Relative abundance (RA) of the top 20 bacteria at the genus level and the remaining bacteria labelled as “others”. In the noninfected rice samples, *Pantoea* was the most dominant whereas in the rice infected by BGR1 and Δ*tssD1*, *Burkholderia* was the most dominant

**TABLE S1** The representative up‐regulated genes clustered in T6SS group_5 in *Burkholderia glumae* BGR1 within rice plants

**TABLE S2** Bacterial strains and plasmids used in this study

**TABLE S3** Oligonucleotide primers used in this study

**TEXT S1** Bacterial strains, plasmids, and growth conditions; bacterial DNA extraction and PCRs; generation of markerless deletion mutants and complementation strains in *Burkholderia* *glumae* BGR1 and its several phenotypic characteristics; and quantitative reverse transcription PCR

## Data Availability

The sequencing data that support the findings of this study are openly available in the NCBI Bioproject database at https://www.ncbi.nlm.nih.gov/bioproject/, accession number PRJNA622913. Sample information is in the NCBI BioSample database at https://www.ncbi.nlm.nih.gov/biosample/, accession numbers SRR11475244, SRR11475245, SRR11475246, SRR11475247, SRR11475248, SRR11475249, SRR11475250, SRR11475251 and SRR11475252.
