# Supplementary figures and images for: Type VI secretion systems of plant‐pathogenic Burkholderia glumae BGR1 play a functionally distinct role in interspecies interactions and virulence
Source: Mol Plant Pathol. 2020 Jul 9;21(8):1055–69. doi: 10.1111/mpp.12966 (PMC7368126; doi:10.1111/mpp.12966)

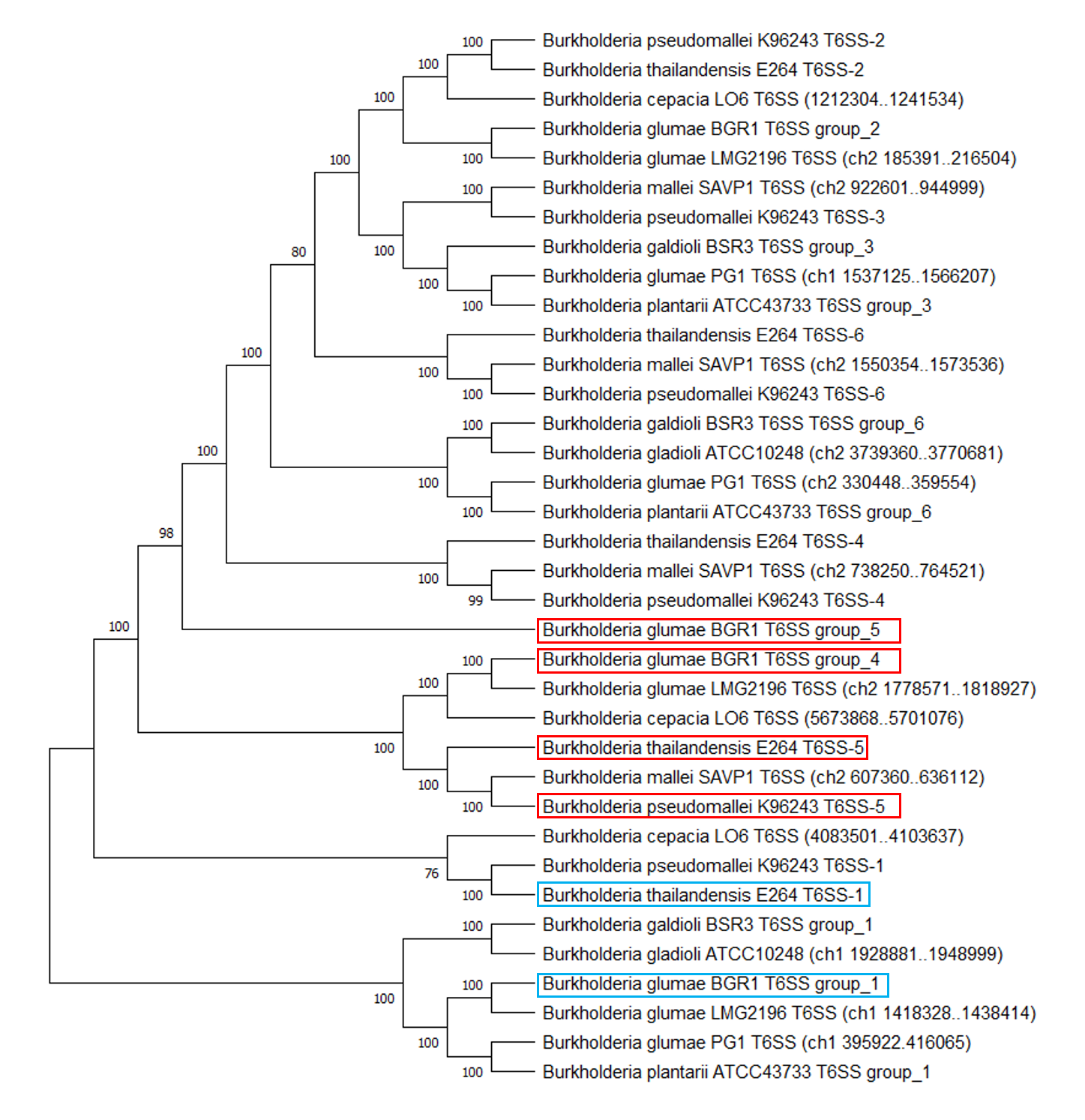

Supplement: Supplementary file 1 — FIGURE S1 Relationship of Burkholderia type VI secretion systems. The relationship of Burkholderia type VI secretion systems was generated using 13 T6SS core components in the T6SS clusters of Burkholderia species. The relationship was inferred using the maximum likelihood method and Jones‐Taylor‐Thornton matrix‐based model. The percentages of replicate trees in which the associated taxa were clustered together in the bootstrap test (1,000 replicates) are shown next to the branches. A discrete gamma distribution was used to model the evolutionary rate differences among sites (five categories [+G, parameter = 4.9003]). Evolutionary analyses were conducted in MEGA X. The eukaryotic targeting system is marked in red and the bacterial targeting system is marked in blue [file MPP-21-1055-s001.tif]

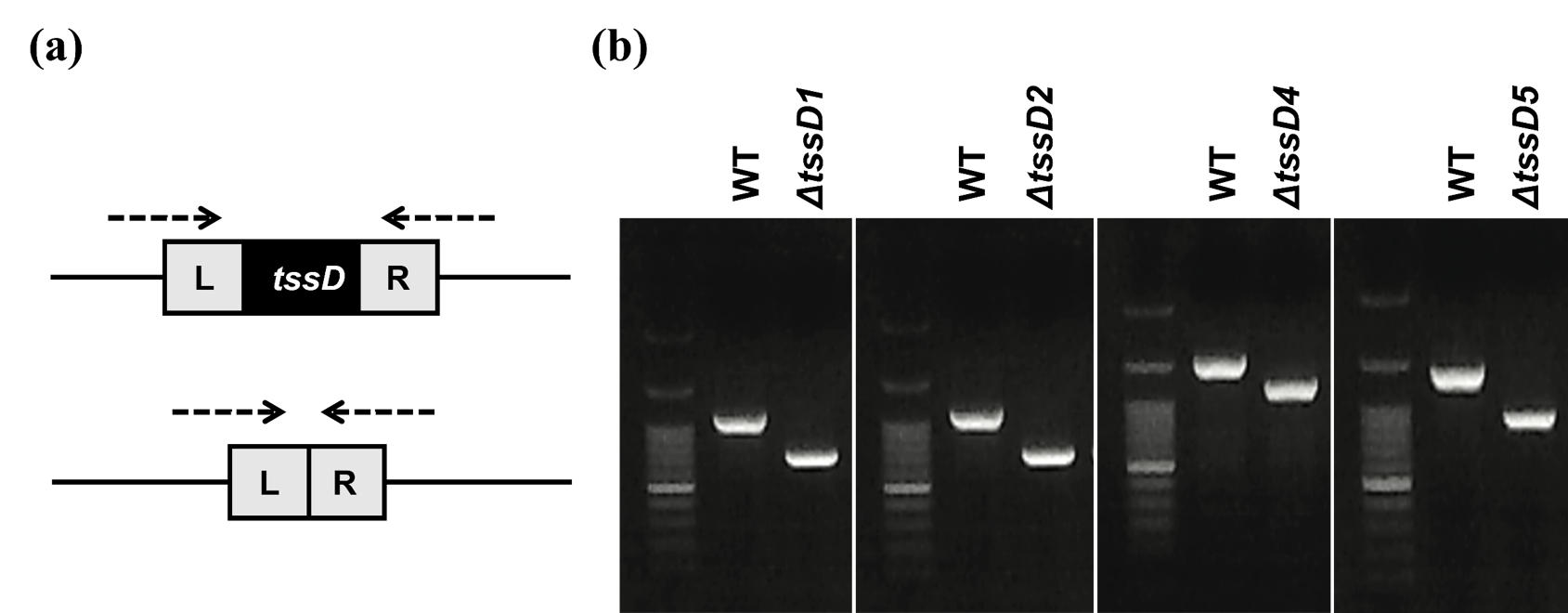

Supplement: Supplementary file 2 — FIGURE S2 Confirmation of tssD mutants using PCR. (a) Deletion mutants were generated and PCR was conducted using primers targeting the 5′‐upstream and 3′‐downstream regions of each tssD region to verify the mutant strains. (b) Agarose gel electrophoresis of the PCR products was performed to distinguish the wild‐type and mutant strains based on different fragment lengths. The PCR product size for each mutant strain was smaller than that obtained from wild‐type BGR1 [file MPP-21-1055-s002.tif]

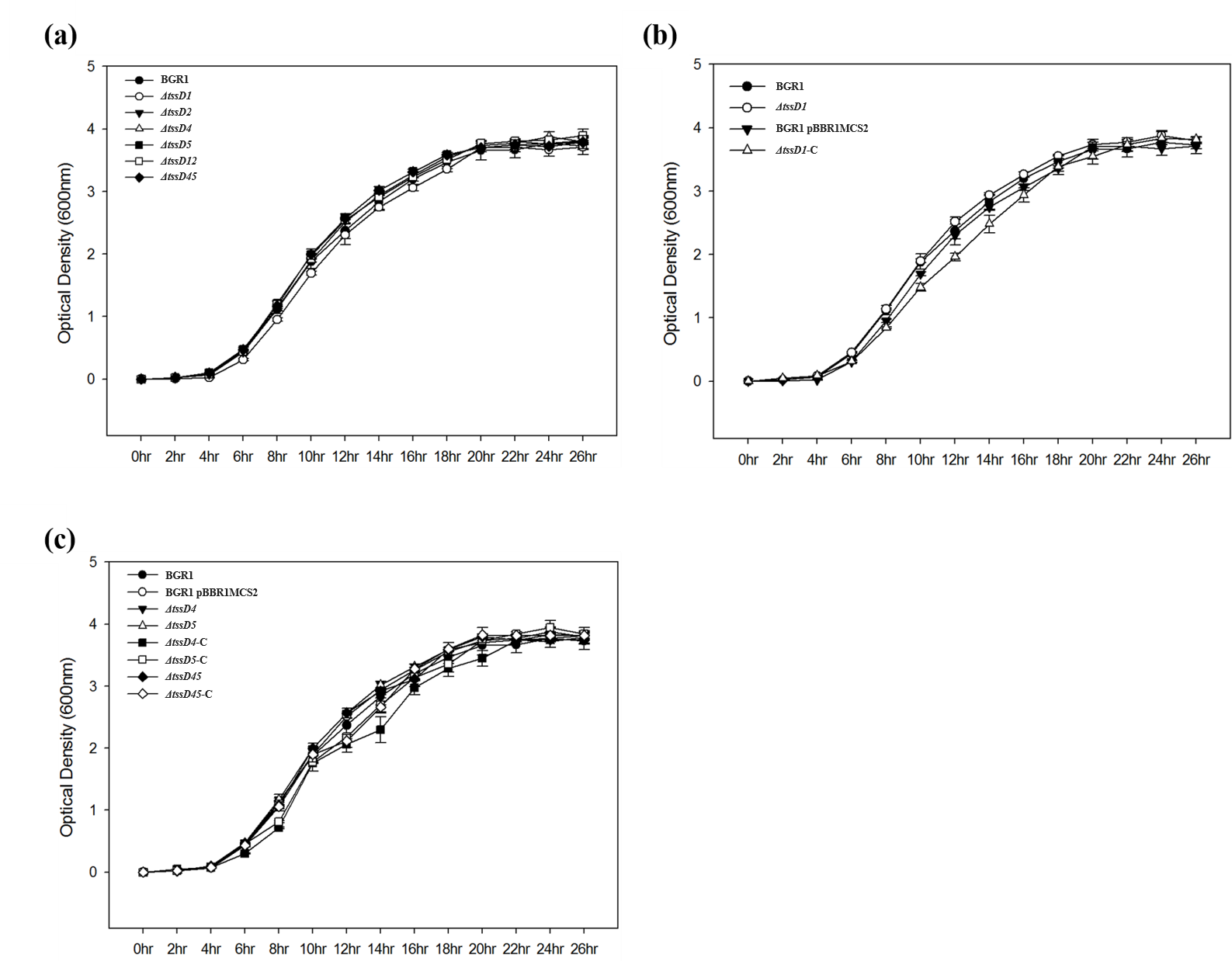

Supplement: Supplementary file 3 — FIGURE S3 Growth curve of Burkholderia glumae BGR1 and tssD mutants. Wild‐type BGR1, tssD single to quadruple deletion mutants, and tssD complementation strains were grown overnight at 37 °C. Overnight bacterial cultures were diluted into fresh Luria broth and then incubated at 37 °C. Bacterial growth was monitored using samples withdrawn every 2 hr and measuring the optical density (600 nm). Compared to the growth pattern of the wild type, no difference was observed in the growth pattern of the mutants. Points indicate the mean values and error bars indicate standard deviations (n = 4) [file MPP-21-1055-s003.tif]

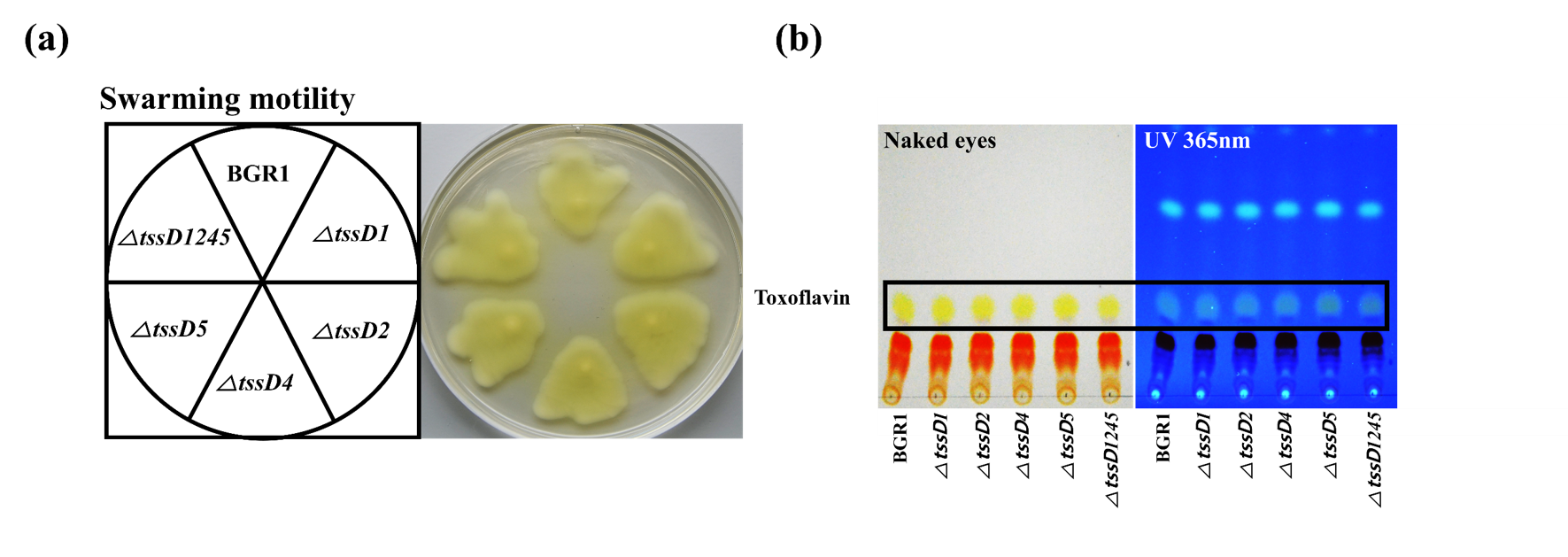

Supplement: Supplementary file 4 — FIGURE S4 Phenotype assay of wild‐type Burkholderia glumae BGR1 and the tssD mutants. (a) Swarming motility by wild‐type BGR1 and the mutants ΔtssD1, ΔtssD2, ΔtssD4, ΔtssD5, and ΔtssD1245. The swarming motility assay was performed on 0.5% agar plates. The image is representative of three independent replicates (n = 3). (b) Biosynthesis of toxoflavin by wild‐type BGR1 and mutants ΔtssD1, ΔtssD2, ΔtssD4, ΔtssD5, and ΔtssD1245. Thin‐layer chromatography analysis was used to detect toxoflavin from the bacterial culture supernatant. The produced toxoflavin was examined visually in daylight and under UV at 365 nm. This is representative of the results from independent experiments with three replicates showing the same pattern [file MPP-21-1055-s004.tif]

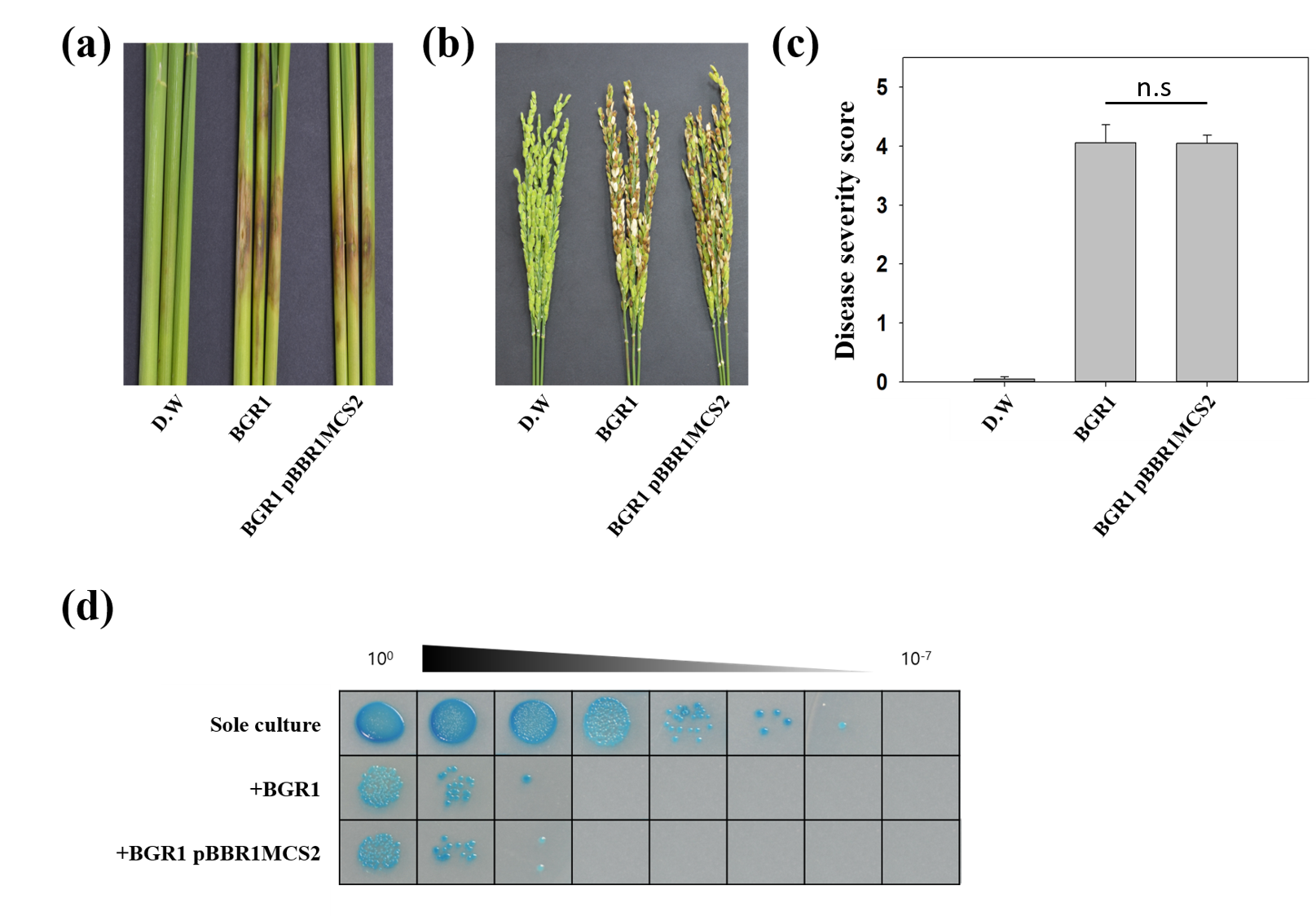

Supplement: Supplementary file 5 — FIGURE S5 Comparison of in vivo pathogenicity assay and antibacterial effect between wild‐type BGR1 and BGR1 pBBR1MCS2. (a) Wild‐type BGR1 and BGR1 containing empty vector were inoculated with 108 cfu/ml to assess the virulence in rice stem at the vegetative stage. (b) Bacterial suspension of wild‐type BGR1 and BGR1 pBBR1MCS2, which contains empty vector of pBBR1MCS2, were inoculated into rice panicles (Oryza sativa) at the reproductive stage to comparison of the virulence. (c) Disease severity on the rice panicles was calculated on a scale of 0 to 5 after inoculating the bacterial suspension. The data are presented as the mean ± SD of three replicates (n = 3). Mean values followed by the same letters are not significantly different according to Tukey’s HSD test (ns, no statistical significance, *p < .05, **p < .01, ***p < .001). At the reproductive and vegetative stage, the disease severity of rice infected with wild‐type BGR1 was not different from that of rice infected with BGR1 pBBR1MCS2. Disease symptoms at 8 days post inoculation. Distilled water was used as the negative control. (d) Antibacterial effects in wild‐type BGR1 and BGR1 pBBR1MCS2. Survival of prey cell was decreased by coculturing with wild‐type BGR1 and BGR1 pBBR1MCS2. Prey cell survival by coculturing with BGR1 pBBR1MCS2 was similar to prey cell survival by coculturing with wild‐type BGR1. This is representative of the results from independent experiments with three replicates showing the same pattern. BGR1 pBBR1MCS2 was used as the negative control of complementation strains. The data were conducted with three replicates [file MPP-21-1055-s005.tif]

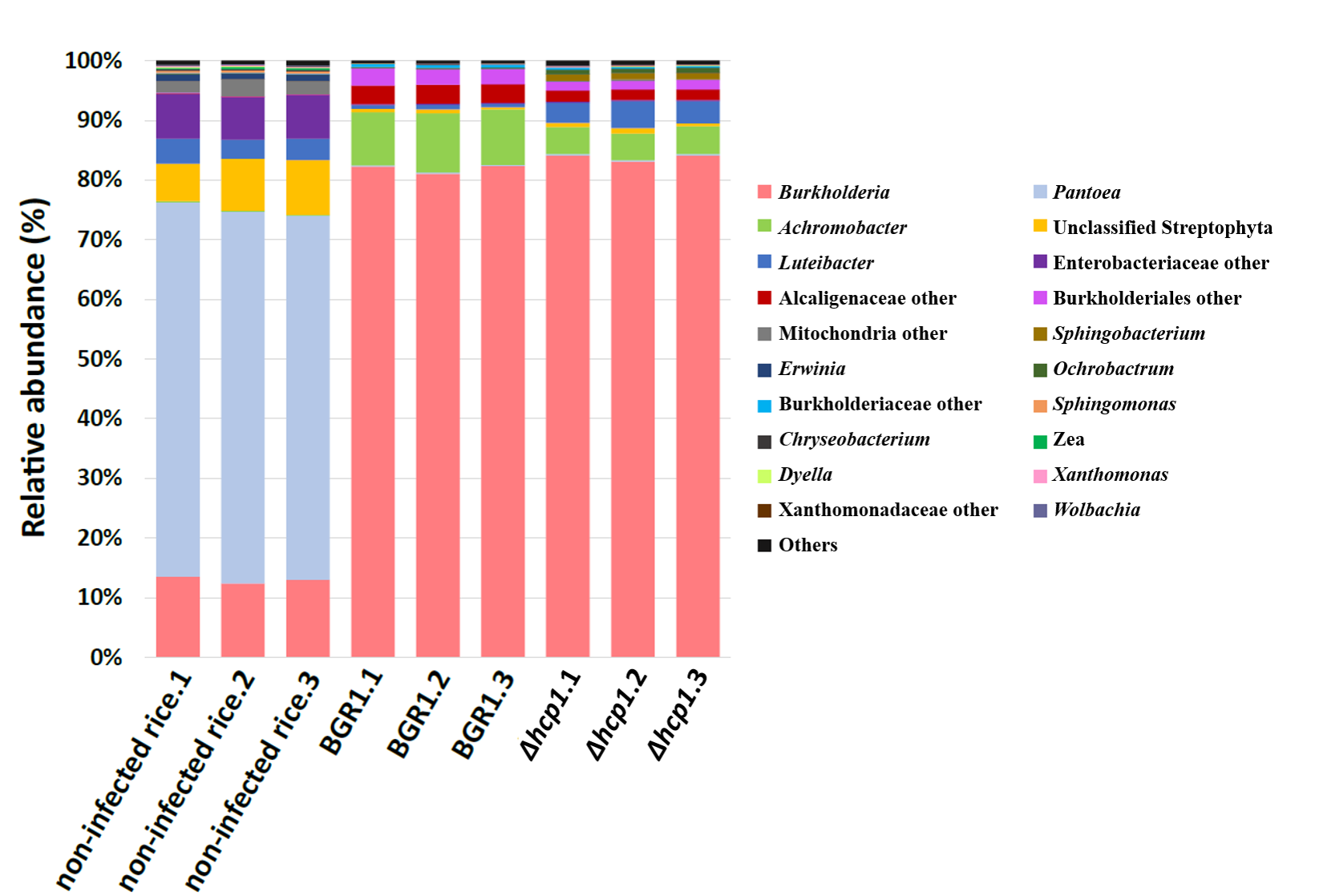

Supplement: Supplementary file 6 — FIGURE S6 Relative abundance of the top 20 genera at the genus level. Relative abundance (RA) of the top 20 bacteria at the genus level and the remaining bacteria labelled as “others”. In the noninfected rice samples, Pantoea was the most dominant whereas in the rice infected by BGR1 and ΔtssD1, Burkholderia was the most dominant [file MPP-21-1055-s006.tif]
