# Supplementary material for: Type VI secretion systems of plant‐pathogenic Burkholderia glumae BGR1 play a functionally distinct role in interspecies interactions and virulence
Source: Mol Plant Pathol. 2020 Jul 9;21(8):1055–69. doi: 10.1111/mpp.12966 (PMC7368126; doi:10.1111/mpp.12966)
Supplement: Supplementary file 7 — TABLE S1 The representative up‐regulated genes clustered in T6SS group_5 in Burkholderia glumae BGR1 within rice plants [file MPP-21-1055-s007.docx]

**Table S1. The representative up-regulated genes clustered in T6SS group_5 in *Burkholderia glumae* BGR1 within rice plants**

| Locus ID | Gene name | log2 (*in vivo* RPKM/*in vitro* RPKM) value | Signature  (p-value < 0.05) |
| --- | --- | --- | --- |
| Bglu_2g07340 | *tssA* | 1.551702947 | p-value < 0.05 |
| Bglu_2g07350 | *tssM* | 2.241604412 | p-value < 0.05 |
| Bglu_2g07370 | *tssK* | 2.269858635 | p-value < 0.05 |
| Bglu_2g07380 | Unknown | 1.598246904 | p-value < 0.05 |
| Bglu_2g07390 | Unknown | 2.269997407 | p-value < 0.05 |
| Bglu_2g07400 | Unknown | 3.385391141 | p-value < 0.05 |
| Bglu_2g07410 | Unknown | 3.865494336 | p-value < 0.05 |
| Bglu_2g07430 | *tssI (vgrG)* | 2.747579459 | p-value < 0.05 |
| Bglu_2g07440 | *tssG* | 2.910926964 | p-value < 0.05 |
| Bglu_2g07450 | *tssF* | 3.283199508 | p-value < 0.05 |
| Bglu_2g07460 | *tssE* | 3.650127672 | p-value < 0.05 |
| Bglu_2g07470 | *tssD (hcp)* | 3.941994377 | p-value < 0.05 |
| Bglu_2g07480 | *tssc* | 5.330264183 | p-value < 0.05 |
