# Supplementary material for: Type VI secretion systems of plant‐pathogenic Burkholderia glumae BGR1 play a functionally distinct role in interspecies interactions and virulence
Source: Mol Plant Pathol. 2020 Jul 9;21(8):1055–69. doi: 10.1111/mpp.12966 (PMC7368126; doi:10.1111/mpp.12966)
Supplement: Supplementary file 8 — TABLE S2 Bacterial strains and plasmids used in this study [file MPP-21-1055-s008.docx]

**Table S2.** Bacterial strains and plasmids used in this study

| **Name** | **Characteristics** | **Source** |
| --- | --- | --- |
| **Bacterial strains** | |  |
| ***B. glumae*** |  |  |
| BGR1 | *Burkholderia glumae* isolate from rice, wild type, Rif^r^ | (Jeong *et al.*, 2003) |
| *ΔtssD1* | BGR1 derivative, deletion of 392 bp within *bglu_1g03910* (*tssD1*) | This study |
| *ΔtssD2* | BGR1 derivative, deletion of 265 bp within *bglu_2g01440* (*tssD2*) | This study |
| *ΔtssD4* | BGR1 derivative, deletion of 399 bp within *bglu_2g11090* (*tssD4*) | This study |
| *ΔtssD5* | BGR1 derivative, deletion of 486 bp within *bglu_2g07470* (*tssD5*) | This study |
| *ΔtssD12* | *bglu_1g03910 (tssD1)* and *bglu_2g01440 (tssD2)* double deletion mutant derived from BGR1 | This study |
| *ΔtssD45* | *bglu_1g11090 (tssD4)* and *bglu_2g007470 (tssD5)* double deletion mutant derived from BGR1 | This study |
| *ΔtssD1245* | *bglu_1g03910 (tssD1), bglu_2g01440 (tssD2), bglu_2g11090 (tssD4), bglu_2g07470 (tssD5)* quadruple deletion mutant derived from BGR1 | This study |
| *ΔtssD1*-C | BGR1 *tssD1* deletion mutant (*ΔtssD1*) containing pB*tssD1* | This study |
| *ΔtssD4*-C | BGR1 *tssD4* deletion mutant (*ΔtssD4*) containing pB*tssD4* | This study |
| *ΔtssD5*-C | BGR1 *tssD5* deletion mutant (*ΔtssD5*) containing pB*tssD5* | This study |
| *ΔtssD45*-C | BGR1 *tssD45* double deletion mutant (*ΔtssD45*) containing pB*tssD4* and pB*tssD5* | This study |
| BGR1 pBBR1MCS2 | BGR1 containing pBBR1MCS2 | This study |
|  |  |  |
| ***E. coli*** |  |  |
| *E. coli* DH5α λpir | F^-^ 80d*lacZ*ΔM15 (*lacZYA-argF*) U169 *recA1* *endA1hsdR17* (rk-, mk+) *phoAsupE44* -*thi-1* *gyrA96* *relA1* | Lab collection |
| *E. coli* S17-1 λpir | *hsdR recA* pro RP4-2 (Tc::Mu; Km::Tn7) (*λ pir*) | (Kalogeraki and Winans *et al.*, 1997) |
|  |  |  |
| **Plasmids** |  |  |
| pK18*mobsacB* | Allelic exchange suicide vector, *sacB* Km^r^ | (Schafer *et al.*, 1994) |
| pK18*tssD1* | For constructing *tssD1* KO mutant, pK18*mobsacB*:: 483bp upstream-downstream of *tssD1* region restricted by EcoRI-HindIII, respectively. | This study |
| pK18*tssD2* | For constructing *tssD2* KO mutant, pK18*mobsacB*:: 667bp upstream-downstream of *tssD2* region restricted by EcoRI-HindIII, respectively. | This study |
| pK18*tssD4* | For constructing *tssD4* KO mutant, pK18*mobsacB*:: 879bp upstream-downstream of *tssD4* region restricted by EcoRI-HindIII, respectively. | This study |
| pK18*tssD5* | For constructing *tssD5* KO mutant, pK18*mobsacB*:: 679bp upstream-downstream of *tssD5* region restricted by EcoRI-HindIII, respectively. | This study |
| pBBR1MCS2 | Broad-host-range plasmid, Km^r^, used to construct complementation strains. | (Kovach *et al.*, 1995) |
| pB*tssD1* | For constructing *ΔtssD1* complementation strain, pBBR1MCS2::CDS of *tssD1* | This study |
| pB*tssD4* | For constructing *ΔtssD4* complementation strain, pBBR1MCS2::CDS of *tssD4* | This study |
| pB*tssD5* | For constructing *ΔtssD5* complementation strain, pBBR1MCS2::CDS of *tssD5* | This study |
| pCRISPomyces-2 | Blue-white screening(*lacZα*) and Apm^r^ | Addgene |
| pBBR1P2::*gfp* | pBBR1MCS2::CDS of *gfp* gene | This study |
| pUBN-GFP-DEST | Template for *gfp* gene amplification | Addgene |
| Abbreviation: Rif^r^, Rifampicin resistance; Km^r^, Kanamycin resistance; Apm^r^, Apramycin resistance; Amp^r^, Ampicillin | | |
