# Supplementary material for: Type VI secretion systems of plant‐pathogenic Burkholderia glumae BGR1 play a functionally distinct role in interspecies interactions and virulence
Source: Mol Plant Pathol. 2020 Jul 9;21(8):1055–69. doi: 10.1111/mpp.12966 (PMC7368126; doi:10.1111/mpp.12966)
Supplement: Supplementary file 9 — TABLE S3 Oligonucleotide primers used in this study [file MPP-21-1055-s009.docx]

| **Table S3.** Oligonucleotide primers used in this study | |  |
| --- | --- | --- |
| Name | Sequence (5'→3') | Use |
| tssD1_LF | AAAAAGAATTCCTTCCAGCTCGACGAACTCTC | To amplify the L fragment of *tssD1* |
| tssD1_LR | AAAGGATCCTTGAGTTCAATCCAGCCCGAG | To amplify the L fragment of *tssD1* |
| tssD1_RF | AAAGGATCCAGAACGACAAGACCTACGCG | To amplify the R fragment of *tssD1* |
| tssD1_RR | AAAAAAAGCTTGCGTGTTCAGGATCGATTCG | To amplify the R fragment of *tssD1* |
| tssD1_UP_F | CTTTCCTGAATCGCTGGATCTC | To confirm the disruption of *tssD1* |
| tssD1_DOWN_R | AAGTCGTTGAGTCCATAGGTCA | To confirm the disruption of *tssD1* |
| tssD2_LF | AAAAAGAATTCACTACCAGGCGAAGTTTTTCCT | To amplify the L fragment of *tssD2* |
| tssD2_LR | AAAGGATCCAGACATTCCACGACCAGCTAAG | To amplify the L fragment of *tssD2* |
| tssD2_RF | AAAGGATCCAGTGGTGTCGCTTTCGTTCT | To amplify the R fragment of *tssD2* |
| tssD2_RR | AAAAAAAGCTTTGCAAGCCAGCCCATGATG | To amplify the R fragment of *tssD2* |
| tssD2_UP_F | GGCTCAATGAGTGGGTGATGA | To confirm the disruption of *tssD2* |
| tssD2_DOWN_R | CTCGTTCGGGTTGACACCTTC | To confirm the disruption of *tssD2* |
| tssD4_LF | AAAAAGAATTCAACTGAAACCGGGTTCGACA | To amplify the L fragment of *tssD4* |
| tssD4_LR | AAAGGATCCCCGGAATTGCCATTGCTTCA | To amplify the L fragment of *tssD4* |
| tssD4_RF | AAAGGATCCGCTACGAGACGATCACCTGG | To amplify the R fragment of *tssD4* |
| tssD4_RR | AAAAAAAGCTTGTTCGATCGCGGCAATCAAC | To amplify the R fragment of *tssD4* |
| tssD4_UP_F | GATGCGATCGCCTCCTACGT | To confirm the disruption of *tssD4* |
| tssD4_DOWN_R | GCTTTCGCACGACCGATGAC | To confirm the disruption of *tssD4* |
| tssD5_LF | AAAAAGAATTCGTTCGTAGCGGGCGATGAG | To amplify the L fragment of *tssD5* |
| tssD5_LR | AAAGGATCCCTTCTCGGGGCTCGTCTGA | To amplify the L fragment of *tssD5* |
| tssD5_RF | AAAGGATCCCCGTCGCTGTTCATGATGCT | To amplify the R fragment of *tssD5* |
| tssD5_RR | AAAAAAAGCTTCATGCGTCAGAAGATCGGCT | To amplify the R fragment of *tssD5* |
| tssD5_UP_F | CGATGCTTCTCGCTGAACTG | To confirm the disruption of *tssD5* |
| tssD5_DOWN_R | CGCGCATTACATCAAGGTCA | To confirm the disruption of *tssD5* |
| pk18_DOWN_R | GTG AAG CTA GCT TAT CGC CAT | To confirm the first crossover in the process of constructing deletion mutant. |
| GFP_HindⅢ_F | AAAAAAGCTTAATGAGTAAAGGAGAAGAAC | Amplifying the *gfp* gene to be cloned in pBBR1MCS2. |
| GFP_BamH1_R | AAAAGGATCCTTACGTTTCTCGTTCAGCT | Amplifying the *gfp* gene to be cloned in pBBR1MCS2. |
| CtssD1_F | AAAAAGAATTC ATGTTAGATATCTATCTCAATTTCGG | Amplifying the *tssD1* fragment to be cloned in pBBR1MCS2. |
| CtssD1_R | AAAGGATCC TCAGACCGCGTAGGTCTTGT | Amplifying the *tssD1* fragment to be cloned in pBBR1MCS2. |
| CtssD4_F | AAAAAGAATTC ATGGCAATTCCGGCCTATATGT | Amplifying the *tssD4* fragment to be cloned in pBBR1MCS2. |
| CtssD4_R | AAAGGATCC TCAGGAACGCTCGTTCCAG | Amplifying the *tssD4* fragment to be cloned in pBBR1MCS2. |
| CtssD5_F | AAAAAGAATTC ATGGATCTGATTCTCTTCCAGCC | Amplifying the *tssD5* fragment to be cloned in pBBR1MCS2. |
| CtssD5_R | AAAGGATCC TCAGACGAGCCCCGAGAAGCT | Amplifying the *tssD5* fragment to be cloned in pBBR1MCS2. |
| pB_UP_F | GACTCACTATAGGGCGAATTG | To confirm *tssD* gene fragments in pBBR1MCS2 |
| pB_DOWN_R | CACACAGGAAACAGCTATGAC | To confirm *tssD* gene fragments in pBBR1MCS2 |
| qPCR_tssD1_F | TCGGGCTGGATTGAACTCAAGT | qRT-PCR analysis to determine the *tssD1* expression level |
| qPCR_tssD1_R | TTTCGTGAACACCATGTCGCTG | qRT-PCR analysis to determine the *tssD1* expression level |
| qPCR_tssD2_F | ACCTCAAGCTGACCATGAACGA | qRT-PCR analysis to determine the *tssD2* expression level |
| qPCR_tssD2_R | AACGAAAGCGACACCACTTCAC | qRT-PCR analysis to determine the *tssD2* expression level |
| qPCR_tssD4_F | GCTCGACAACGTCAAGATCGTG | qRT-PCR analysis to determine the *tssD4* expression level |
| qPCR_tssD4_R | CGTCCTTGTACGACCAGGTGAT | qRT-PCR analysis to determine the *tssD4* expression level |
| qPCR_tssD5_F | CTGCGTGTAGGTCCAGATGACT | qRT-PCR analysis to determine the *tssD5* expression level |
| qPCR_tssD5_R | GCGGCAAGACGATGAACATCAT | qRT-PCR analysis to determine the *tssD5* expression level |
| qPCR_16s_F | TCC TGA GGG CTA ATA TCC TTC G | qRT-PCR analysis of 16s rRNA used as internal control |
| qPCR_16s_R | ACC AAT GCA GTT CCC AGG TTG A | qRT-PCR analysis of 16s rRNA used as internal control |
| * Underlined sequence in the primers indicates restriction enzyme targeted sequences | | |
