# Supplementary material for: Type VI secretion systems of plant‐pathogenic Burkholderia glumae BGR1 play a functionally distinct role in interspecies interactions and virulence
Source: Mol Plant Pathol. 2020 Jul 9;21(8):1055–69. doi: 10.1111/mpp.12966 (PMC7368126; doi:10.1111/mpp.12966)
Supplement: Supplementary file 10 — TEXT S1 Bacterial strains, plasmids, and growth conditions; bacterial DNA extraction and PCRs; generation of markerless deletion mutants and complementation strains in Burkholderia glumae BGR1 and its several phenotypic characteristics; and quantitative reverse transcription PCR [file MPP-21-1055-s010.docx]

**Bacterial strains, plasmids, and growth conditions**

All bacterial strains and plasmids used in this study are described in Table S1. The wild-type BGR1, mutant strains, and *E. coli* were cultured on Luria-Bertani (LB) agar and in broth medium at 37℃ with shaking at 200 rpm. The growth curve of bacterial strains was determined every 2 h by measuring the optical density (600 nm) using a UV-1800 spectrophotometer (Shimadzu, Kyoto, Japan). When required, LB media were supplemented with antibiotics at the following concentrations: rifampicin, 100 μg/mL; kanamycin, 50 μg/mL; tetracycline, 10 μg/mL; and apramycin, 50 μg/mL.

**Bacterial DNA extraction and polymerase chain reactions**

Bacterial genomic DNA was extracted using the Wizard^®^ Genomic DNA Purification Kit (Promega, Madison, WI, USA) following the manufacturer’s protocol. Plasmid DNA was isolated using the Dokdo-Prep^TM^ Plasmid DNA Mini-prep kit (Elpis Biotech, Daejeon, South Korea). Polymerase chain reaction (PCR) was performed using the Solg^TM^ *Pfu-X* DNA polymerase (Solgent, Daejeon, South Korea) to prepare the fragments to be cloned in pK18*tssDs*, pBBR1MCS2::*gfp,* and pB*tssDs*. PCR was performed in 50 μL total volumes following the protocol. The PCR conditions were as follows: 2 min at 95℃, followed by 35 cycles of 30 s of 95℃, 30 s at the annealing temperature, 30 s/kb at 72℃. The used primers are listed in Table S2. PCR was performed to verify the mutant strain generated above. PCR was carried out using a Sure Cycler 8800 Thermal cycler (Agilent Technologies, Santa Clara, CA, USA).

**Generation of markerless deletion mutants and complementation strains in *B. glumae* BGR1 and its several phenotypic characteristics**

For constructing markerless deletion mutants, the plasmid pK18*tssD*s, harbouring portions of the *tssD* upstream (L fragment) and downstream region (R fragment), was prepared. To prepare pK18*tssD*s, the L and R fragments were amplified using gDNA and the corresponding primer sets listed in Table S1. The L and R fragments were digested with BamHI (NEB, Ipswich, MA, USA) and ligated. The ligated LR fragment and pK18*mobsacB* were digested with EcoRI and HindIII and then ligated. Competent *E. coli* DH5α cells were transformed with the recombinant plasmid, pK18*tssD*s, and cells harbouring pK18*tssD*s were then selected in kanamycin-containing media. pK18*tssDs* were transformed into *E. coli* S17-1. pK18*tssDs* were transformed into *B. glumae* BGR1 by conjugation through coculture with *E. coli* S17-1 on LB agar. Then, the first crossover was selected on rifampicin (100 μg/mL) and kanamycin (60 μg/mL)-containing media. Three days later, the first crossover of colonies grown on the plate was picked and confirmed by PCR using the primer sets of tssD_UP_F and pk18_DOWN_R. The selected cells underwent a second crossover by two subcultures in LB broth every 12 h. The second crossover in the cells was selected on LB agar containing 30% sucrose and rifampicin (100 μg/mL). Finally, single *tssD* deletion mutants, with deletion of *tssD1*, *tssD2*, *tssD4*, and *tssD5* gene, respectively, were confirmed by PCR using the primer sets for tssD_UP_F and tssD_DOWN_R. Double *tssD* deletion mutants were constructed based on single *tssD* deletion mutants using the above-mentioned methods. To construct quadruple deletion mutants, triple *tssD* deletion mutants were generated from double *tssD* deletion mutants using the above-mentioned methods. Quadruple *tssD* deletion mutants were also constructed using the above-mentioned methods based on triple *tssD* deletion mutants. For complementation, the entire open reading frame of *tssD1*, *tssD4*, and *tssD5* genes was amplified by PCR using the primer sets of CtssD_F and CtssD_R, and the amplified fragments were cloned into the broad-host-range expression vector, pBBR1MCS2. The cloned vectors (pB*tssD*s) were then conjugated into each *tssD* mutant strain by co-culturing with S17-1. Finally, complementation strains of single *tssD* deletion mutants were selected on rifampicin (100 μg/mL) and kanamycin (60 μg/mL)-containing media and were confirmed by PCR using primer sets of CtssDs_F and pBBR1MCS2_dR. For constructing the complementation strains of double *tssD* deletion mutants, *ΔtssD45*, both the transformed S17-1 containing pB*tssD4* and the transformed S17-1 containing pB*tssD5* were co-cultured with double *tssD* deletion mutants for simultaneous conjugation on LB agar plate. The complementation strains of double *tssD* deletion mutants were selected on rifampicin (100 μg/mL) and kanamycin (60 μg/mL)-containing media. Colonies grown on the plate were picked randomly and confirmed by PCR using the primer sets of CtssDs_F and pB_UP_F, simultaneously. Several phenotypic characteristics assessed including bacterial motility, by which *B. glumae* cells reach the proper infection site, and a thin-layer chromatography assay of toxoflavin, was performed following the methods described by Lee *et al.*, (2016).

**Quantitative RT-PCR**

For assessing the expression levels of the four *tssD* genes depending on the presence of prey, cultures of *B. glumae* BGR1 and co-cultures of *B. glumae* BGR1 with *E. coli* as the prey on LB agar plate for 4 h were used. The cells were gathered on the LB agar plate. Total RNA was extracted using the RNeasy Mini Kit (Qiagen, Hilden, Germany). The cDNA was synthesized from 500 ng of total RNA with random hexamers using SuperiorScript III cDNA synthesis kit (Enzynomics, Daejeon, South Korea). qRT-PCR analysis was performed using Rotor-gene Q (Qiagen, Hilden, Germany). All samples were analysed in 20 μL reaction mixtures containing 10 μL of TOPreal^TM^ qPCR 2× PreMIX, 1 μL of cDNA, 1 μL of qPCR_tssD_F, 1 μL of qPCR_tssD_R, and 7 μL of DW. The relative gene expression levels were determined by the 2^-ΔΔCt^ method with the 16S ribosomal RNA gene as the reference gene.
